# Attenuated cerebellar phenotypes in *Inpp4a* truncation mutants with preserved phosphatase activity

**DOI:** 10.1242/dmm.050169

**Published:** 2023-07-28

**Authors:** Dang Minh Tran, Nozomu Yoshioka, Norihisa Bizen, Yukiko Mori-Ochiai, Masato Yano, Shogo Yanai, Junya Hasegawa, Satoshi Miyashita, Mikio Hoshino, Junko Sasaki, Takehiko Sasaki, Hirohide Takebayashi

**Affiliations:** ^1^ Division of Neurobiology and Anatomy, Graduate School of Medical and Dental Sciences, Niigata University, Niigata 951-8510, Japan; ^2^ Department of Biochemical Pathophysiology, Medical Research Institute, Tokyo Medical and Dental University (TMDU), Tokyo 113-8510, Japan; ^3^ Department of Biochemistry and Cellular Biology, National Institute of Neuroscience, National Center of Neurology and Psychiatry (NCNP), Tokyo 187-8502, Japan; ^4^ Department of System Pathology for Neurological Disorders, Brain Research Institute, Niigata University, Niigata 9518585, Japan; ^5^ Center for Coordination of Research Facilities (CCRF), Niigata University, Niigata 951-8510, Japan

**Keywords:** Phosphoinositide, Cerebellar atrophy, Inositol polyphosphate-4-phosphatase type I, Pain-induced epilepsy, Neurodegeneration, Mouse model

## Abstract

Phosphoinositides (PIPs) act as intracellular signaling molecules that regulate various cellular processes. Abnormalities in PIP metabolism cause various pathological conditions, including neurodegenerative diseases, cancer and immune disorders. Several neurological diseases with diverse phenotypes, such as ataxia with cerebellar atrophy or intellectual disability without brain malformation, are caused by mutations in *INPP4A*, which encodes a phosphoinositide phosphatase. We examined two strains of *Inpp4a* mutant mice with distinct cerebellar phenotypes: the *Inpp4a^ΔEx1,2^* mutant exhibited striatal degeneration without cerebellar atrophy, and the *Inpp4a^ΔEx23^* mutant exhibited a severe striatal phenotype with cerebellar atrophy. Both strains exhibited reduced expression of Inpp4a mutant proteins in the cerebellum. N-terminal-truncated Inpp4a proteins were expressed from the *Inpp4a^ΔEx1,2^* allele by alternative translation initiation and had phosphatase activity for PI(3,4)P_2_, whereas the Inpp4a mutant protein encoded by *Inpp4a^ΔEx23^* completely lacked phosphatase activity. Our results indicate that the diverse phenotypes observed in *Inpp4a*-related neurological diseases could be due to the varying protein expression levels and retained phosphatase activity in different Inpp4a variants. These findings provide insights into the role of INPP4A mutations in disease pathogenesis and may help to develop personalized therapy.

## Teaser

**Summary:** Our study reveals diverse cerebellar phenotypes of *Inpp4a* mutant mouse lines and could provide insights into the role of *INPP4A* mutations in neurodegenerative disease pathogenesis.

## INTRODUCTION

Phosphoinositides (PIPs) are phosphorylated forms of phosphatidylinositol that are present at relatively low levels within cells. These are unique phospholipids because they can be modified rapidly by headgroup phosphorylation/dephosphorylation by dozens of kinases and phosphatases to transiently generate (or remove) membrane-targeting signals at particular intracellular locations (Balla, 2013; Sasaki et al., 2009). PIPs regulate various cellular processes, including cytoskeletal remodeling, membrane trafficking and ion channel activity (Echard, 2012; Nilius et al., 2008; Roth, 2004; Zolles et al., 2006). Abnormal metabolism of PIPs is involved in various physiological and pathological conditions, including developmental defects (Wu et al., 2020), cancer (Bunney and Katan, 2010), neurological disease (Volpatti et al., 2019; Waugh, 2015) and immune disorders (Aich et al., 2012; Nigorikawa et al., 2015).

INPP4A catalyzes the removal of the 4′-phosphate of phosphatidylinositol 3,4-bisphosphate [PI(3,4)P_2_] and phosphatidylinositol 3,4,5-trisphosphate [PI(3,4,5)P_3_]. PI(3,4)P_2_ is a phosphoinositide 3-kinase-generated lipid second messenger. INPP4A is widely and highly expressed in the brain, heart and skeletal muscle (Norris et al., 1995). Ligand binding to growth factor receptors, such as Trk (also known as NTRK) receptors, activates phosphoinositide 3-kinase, which then generates PI(3,4,5)P_3_ from PI(4,5)P_2_ (Vanhaesebroeck et al., 2010). Subsequently, SHIP1 and SHIP2 (also known as INPP5D and INPPL1, respectively) generate PI(3,4)P_2_ from PI(3,4,5)P_3_ (Hawkins and Stephens, 2016). PI(3,4)P_2_ regulates neurite and dendrite development, and the phosphoinositide metabolism of PI(3,4)P_2_ is crucial for neuronal development and the proper function of synapses in the nervous system (Zhang et al., 2017). Additionally, some PIPs metabolism enzyme genes are tumor suppressor genes, such as phosphatase and tensin homolog (*PTEN*) and *INPP4B*. PTEN catalyzes removal of the 3′-phosphate of PI(3,4)P_2_ and PI(3,4,5)P_3_ (Fukumoto et al., 2017), and the N-terminal domain of PTEN binds to PIP_2_ (Rahdar et al., 2009). INPP4B is involved in DNA repair (Sun et al., 2020) and is a potential biomarker for the resistance of cancer cells to radiotherapy (Kim et al., 2012). Spontaneous mutation of the *Inpp4a* gene in *weeble* mutant mice results in postnatal cerebellar and striatal neuronal degeneration (Nystuen et al., 2001). Neurodegeneration in the striatum of *Inpp4a* knockout (KO) mice lacking the first and second coding exons (exons 1 and 2) was caused by excitotoxicity (Sasaki et al., 2010). Studies have reported human diseases associated with *INPP4A* mutations [Online Mendelian Inheritance in Man (OMIM) 600916] (Banihashemi et al., 2020; Hecher et al., 2023; Najmabadi et al., 2011; Özkan Kart et al., 2023; Sheffer et al., 2015); however, different mutations result in different disease phenotypes. A nonsense mutation, c.115 C>T p.Gln39X, in the conserved N-terminal domain of INPP4A leads to intellectual disability without brain malformation (Banihashemi et al., 2020), while homozygous genomic deletion of 1770 bp within the *INPP4A* gene causes myoclonic epilepsy, microcephaly and atrophy of the cerebellum (Sheffer et al., 2015). Thus, there is diversity in the neuropathological phenotypes caused by different *INPP4A* mutations, and the underlying molecular mechanisms are unknown.

To understand the heterogeneous brain phenotypes of *Inpp4a* mutants, we examined two strains of *Inpp4a* mutant mice that exhibit different phenotypes. One strain has cerebellar atrophy, and the other does not. *Inpp4a* mutants lacking exons 1 and 2 (*Inpp4a^ΔEx1,2^* KO mice) exhibited striatal degeneration but an intact cerebellum. In contrast, *Inpp4a* mutants lacking exon 23, which encodes a C-terminal phosphatase domain (*Inpp4a^ΔEx23^* KO mice), exhibited striatal degeneration and cerebellar atrophy. We demonstrate that the N-terminal-truncated Inpp4a protein expressed in *Inpp4a^ΔEx1,2^* KO mice has phosphatase activity, which may account for the attenuated cerebellar phenotypes in the *Inpp4a^ΔEx1,2^* KO mice and phenotypic diversity of the *Inpp4a* mutant phenotypes.

## RESULTS

### Diversity of cerebellar phenotypes in the two *Inpp4a* KO mouse lines

**Fig. 1.**
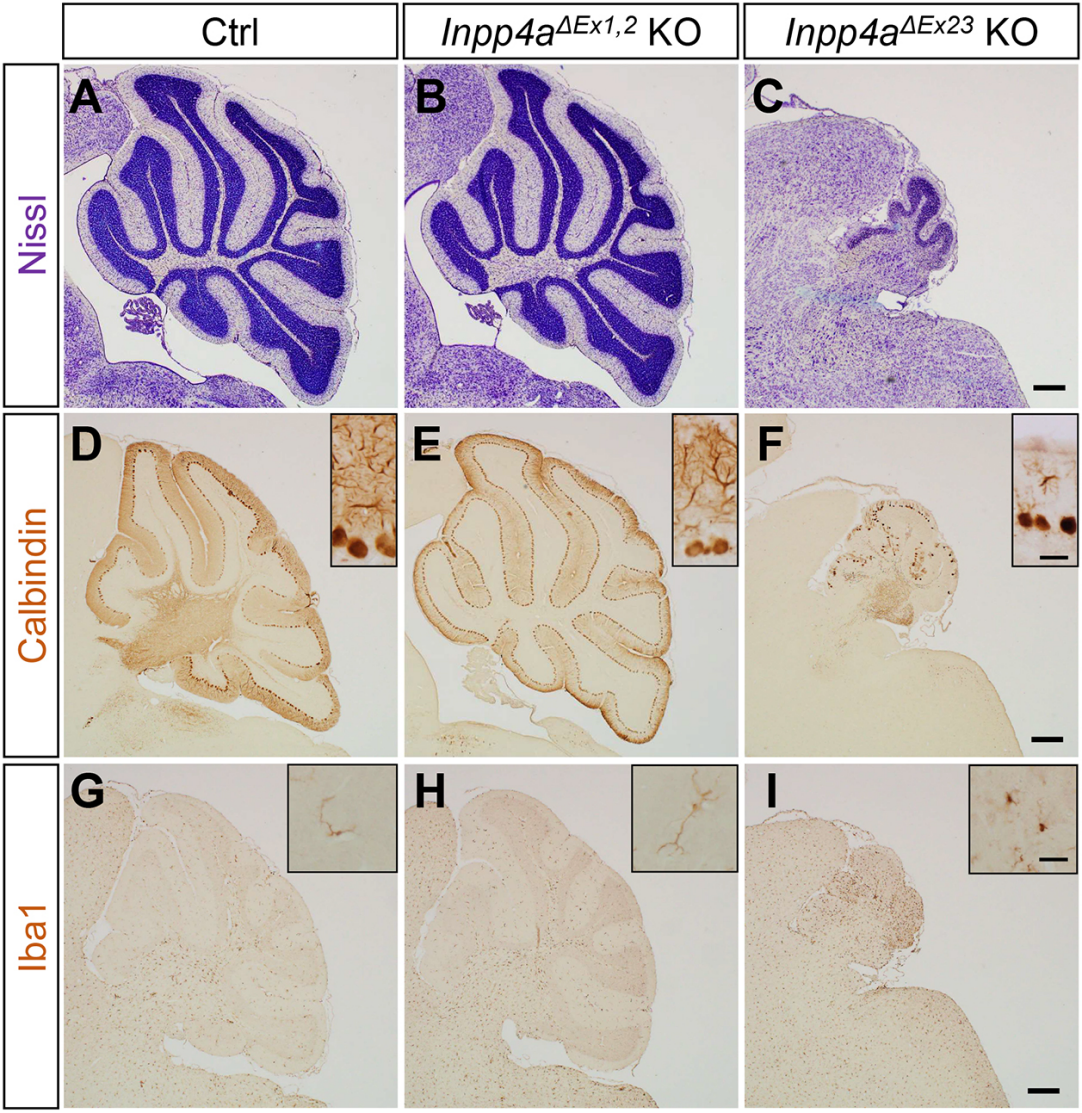
**Histological analysis of the cerebellum in *Inpp4a^ΔEx1,2^* and *Inpp4a^ΔEx23^* mutant mice.** (A–C) Parasagittal Nissl-stained cerebellum sections of control (Ctrl), *Inpp4a^ΔEx1,2^* knockout (KO) and *Inpp4a^ΔEx23^* KO mice at 3 weeks of age (*n*=3 mice, each genotype). (D–F) Calbindin immunohistochemistry (IHC) in parasagittal cerebellum sections of Ctrl, *Inpp4a^ΔEx1,2^* KO and *Inpp4a^ΔEx23^* KO mice at 3 weeks of age (*n*=3 mice, each genotype). (G–I) Iba1 IHC in parasagittal cerebellum sections of Ctrl, *Inpp4a^ΔEx1,2^* KO and *Inpp4a^ΔEx23^* KO mice at 3 weeks of age (*n*=3 mice, each genotype). Insets in calbindin and Iba1 rows show the shape of Purkinje cells and microglia, respectively. Scale bars: 200 μm; 50 µm (insets).

**Fig. 2.**
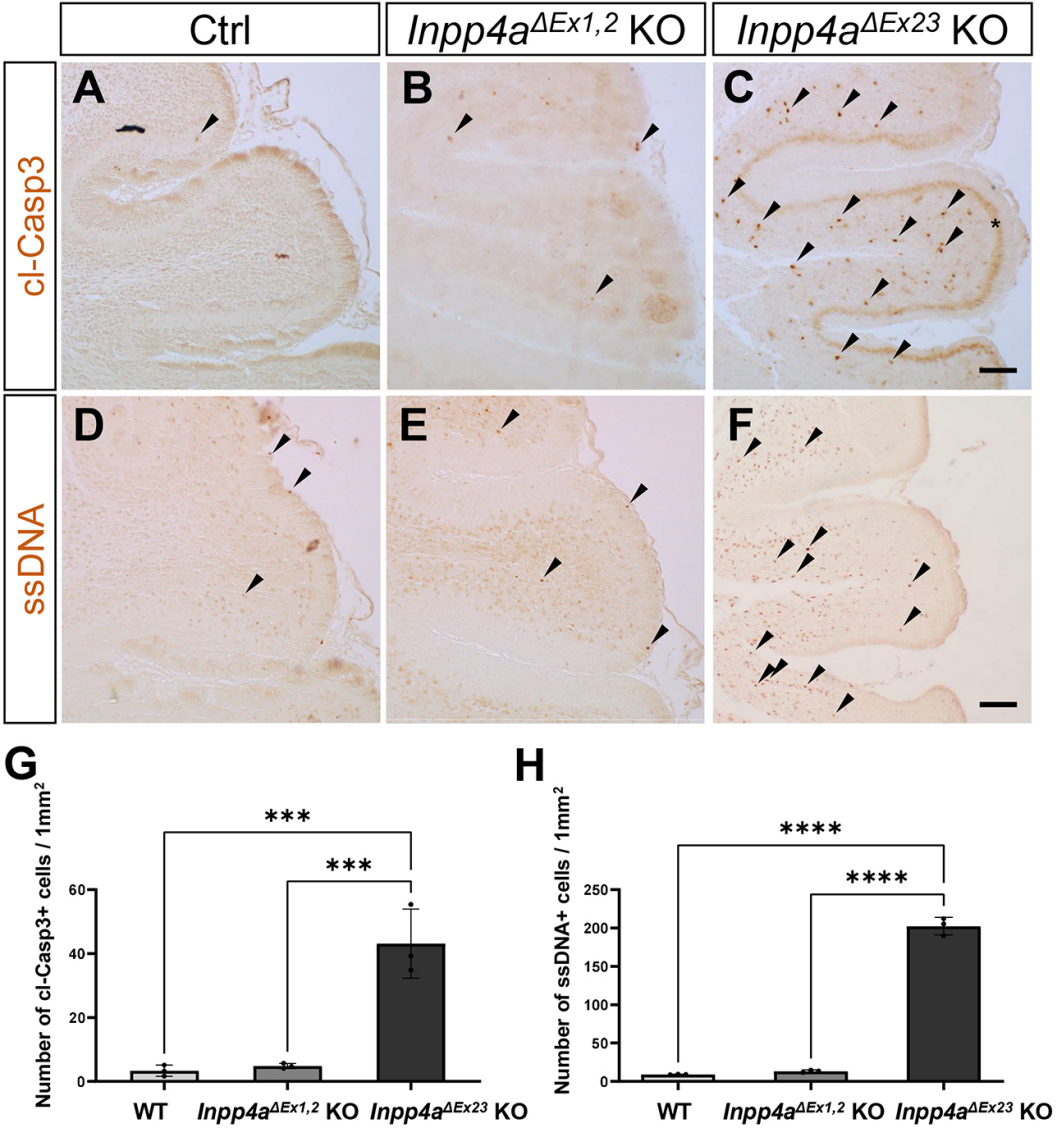
**Increased apoptotic cells in the *Inpp4a^ΔEx23^* KO cerebellum.** (A–C) Cleaved caspase-3 (cl-Casp3) IHC in parasagittal cerebellum sections of Ctrl, *Inpp4a^ΔEx1,2^* KO and *Inpp4a^ΔEx23^* KO mice at 1 week of age (*n*=3 mice, each genotype). Arrowheads indicate cl-Casp3-positive cells. Asterisk indicates cl-Casp3-positive signal in the Purkinje cell layer. (D–F) Single-stranded DNA (ssDNA) IHC in parasagittal cerebellum sections of Ctrl, *Inpp4a^ΔEx1,2^* KO and *Inpp4a^ΔEx23^* KO mice at 1 week of age (*n*=3 mice, each genotype). Arrowheads indicate ssDNA-positive cells. (G) Quantification of cl-Casp3-positive cells per 1 mm^2^ in A–C. (H) Quantification of ssDNA-positive cells per 1 mm^2^ in D–F. All values in the graphs are presented as means±s.d. ***P<0.001, ****P<0.0001 (one-way ANOVA). Sample calculation and tests for outliers were not performed. Scale bars: 100 μm.

*Inpp4a^ΔEx1,2^* KO mice exhibited severely disordered involuntary movement, including limb hyperkinesia, opisthotonos and dystonia (Movie 1) (Sasaki et al., 2010). *Inpp4a^ΔEx23^* KO mice showed cerebellar atrophy and ataxic gait (Movie 2), similar to *weeble* mice (Nystuen et al., 2001). The cerebellum in *Inpp4a^ΔEx1,2^* KO mice was of standard size, compared with a relatively small cerebellum in *Inpp4a^ΔEx23^* KO mice (Fig. S1A,D). The brain and body weights of the *Inpp4a* mutants were smaller than those of wild-type mice (Fig. S1B,C,E,F). Histological analysis confirmed that wild-type cerebellum and *Inpp4a^ΔEx1,2^* KO cerebellum were of indistinguishable size (Fig. 1A,B) and that the *Inpp4a^ΔEx23^* KO cerebellum showed apparent atrophy (Fig. 1C). *Inpp4a^ΔEx23^* KO mice exhibited Purkinje cell loss (Fig. 1F) and activation of microglia (Fig. 1I), which were not present in *Inpp4a^ΔEx1,2^* KO or wild-type mice (Fig. 1D,E,G,H). In addition, *Inpp4a^ΔEx1,2^* KO mice exhibited pain-induced epilepsy (Movie 3), whereas *Inpp4a^ΔEx23^* KO mice did not. These data indicate phenotypic diversity in the cerebellum between *Inpp4a^ΔEx1,2^* and *Inpp4a^ΔEx23^* KO mice. Purkinje cell degeneration was observed in the *Inpp4a^ΔEx23^* KO cerebellum at the postnatal stage (Fig. S2) as previously reported in *weeble* mice (Nystuen et al., 2001). Next, we performed immunohistochemistry (IHC) using antibodies against the apoptotic markers cleaved caspase-3 (cl-Casp3) and single-stranded DNA (ssDNA). There was remarkable upregulation of cl-Casp3 and ssDNA signals in the cerebellum of *Inpp4a^ΔEx23^* KO mice but not in that of *Inpp4a^ΔEx1,2^* KO mice (Fig. 2A–H). The apoptotic cells were mainly in the granule cell layer of the cerebellum at postnatal day (P)7 (Fig. 2C,F), and cl-Casp3-positive Purkinje cells were present in the *Inpp4a^ΔEx23^* KO cerebellum (Fig. 2C). Despite the almost intact cerebellum in *Inpp4a^ΔEx1,2^* mice, neurodegeneration was present in the striatum (Sasaki et al., 2010). We also confirmed activation of microglia and astrocytes in the striatum of both *Inpp4a^ΔEx1,2^* and *Inpp4a^ΔEx23^* KO mice (Fig. S3A–G), with the activation being more severe in *Inpp4a^ΔEx23^* KO mice (Fig. S3H).

### Alternative exon use of *Inpp4a* transcripts in the cerebellum

**Fig. 3.**
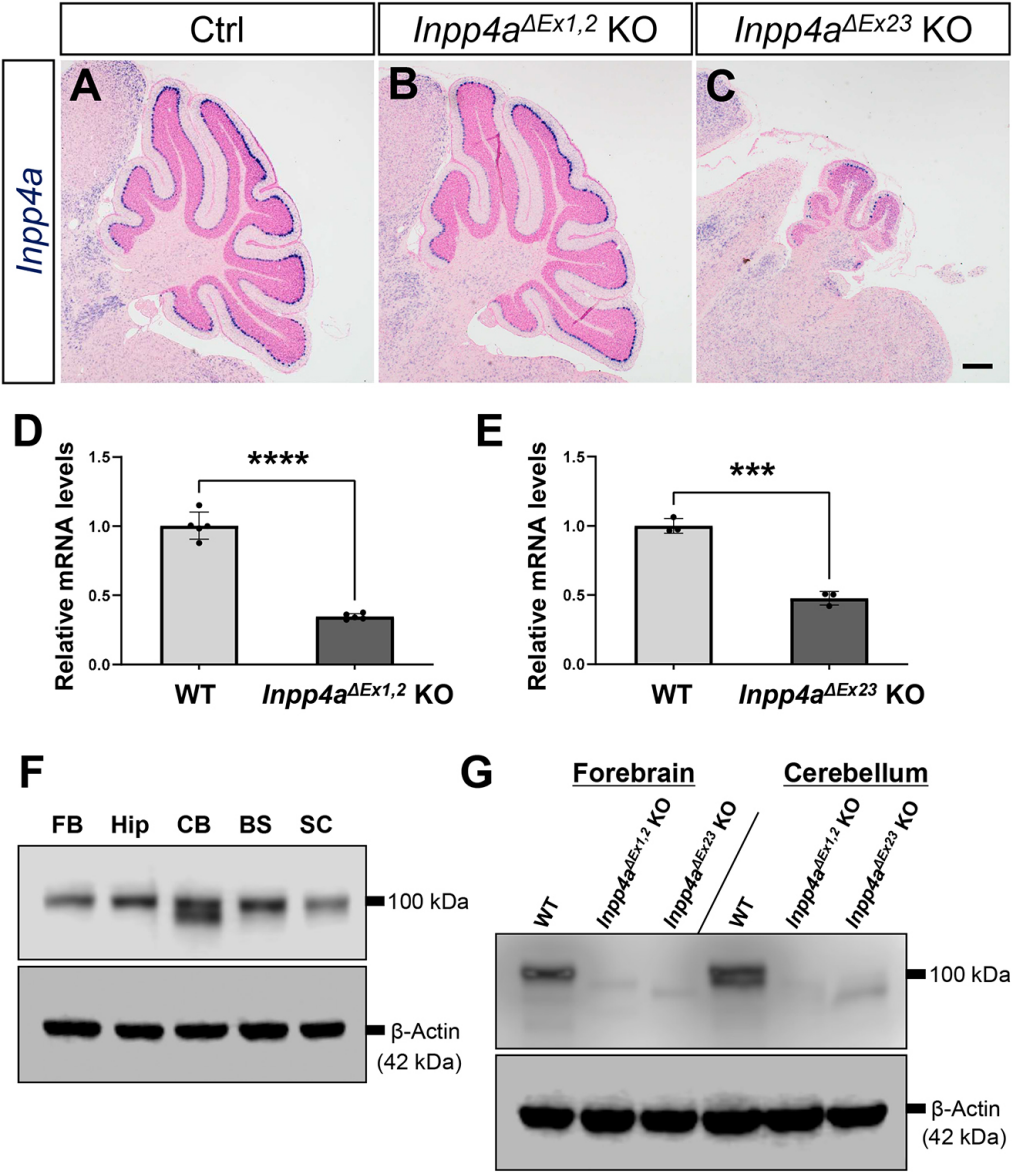
**Expression of *Inpp4a* mRNA and Inpp4a protein.** (A–C) *Inpp4a* ISH in parasagittal cerebellum sections of Ctrl, *Inpp4a^ΔEx1,2^* KO and *Inpp4a^ΔEx23^* KO mice at 3 weeks of age (*n*=3 mice, each genotype). Scale bar: 200 µm. (D) Quantitative PCR (qPCR) data on *Inpp4a* mRNA in the cerebellum of Ctrl and *Inpp4a^ΔEx1,2^* KO mice at 2 weeks of age (*n*=4 mice, each genotype). All values in the graphs are presented as means±s.d. ****P<0.0001 (unpaired two-tailed Student's *t*-test). (E) qPCR of *Inpp4a* mRNA in the cerebellum of Ctrl and *Inpp4a^ΔEx23^* KO mice at 3 weeks of age (*n*=3 mice, each genotype). All values in the graphs are presented as means±s.d. ***P<0.001 (unpaired two-tailed Student's *t*-test). (F) Western blotting by rat monoclonal anti-Inpp4a antibody in the forebrain (FB), hippocampus (Hip), cerebellum (CB), brainstem (BS) and spinal cord (SC) of 3-week-old wild-type (WT) mice. There were two Inpp4a bands only in the cerebellum. β-Actin is the internal control (*n*=3 mice). (G) Western blotting by rat monoclonal anti-Inpp4a antibody in the forebrain and cerebellum of WT, *Inpp4a^ΔEx1,2^* KO and *Inpp4a^ΔEx23^* KO mice at 3 weeks of age (*n*=3 mice, each genotype). Inpp4a bands were significantly diminished in both *Inpp4a^ΔEx1,2^* KO and *Inpp4a^ΔEx23^* KO mice. β-Actin was used as an internal control.

We performed *in situ* hybridization (ISH) to investigate *Inpp4a* expression in wild-type mice and the two lines of *Inpp4a* KO mice. In wild-type mice, *Inpp4a* was widely expressed throughout the brain and cerebellum with very high levels in Purkinje cells (Fig. 3A) (Nystuen et al., 2001). *Inpp4a* mRNA was also detected in the brain of *Inpp4a^ΔEx1,2^* and *Inpp4a^ΔEx23^* KO mice (Fig. 3B,C); however, quantitative PCR (qPCR) data indicated a significant decrease in the level of *Inpp4a* mRNA compared with that in the brain of wild-type mice (Fig. 3D,E). Next, we examined the expression of Inpp4a protein in each area of the wild-type central nervous system by western blotting and detected two isoforms only in the cerebellum (Fig. 3F). We also investigated the expression of Inpp4a protein in the forebrain and cerebellum of wild-type, *Inpp4a^ΔEx1,2^* KO and *Inpp4a^ΔEx23^* KO mice (Fig. 3G). As expected from the reduced mRNA levels, we observed dramatically reduced levels of mutant Inpp4a protein in the forebrain and cerebellum of *Inpp4a^ΔEx1,2^* KO and *Inpp4a^ΔEx23^* KO mice (Fig. 3G). We further confirmed the Inpp4a signals in the Purkinje cells of wild-type, *Inpp4a^ΔEx1,2^* KO and *Inpp4a^ΔEx23^* KO mice using IHC (Fig. S4).

**Fig. 4.**
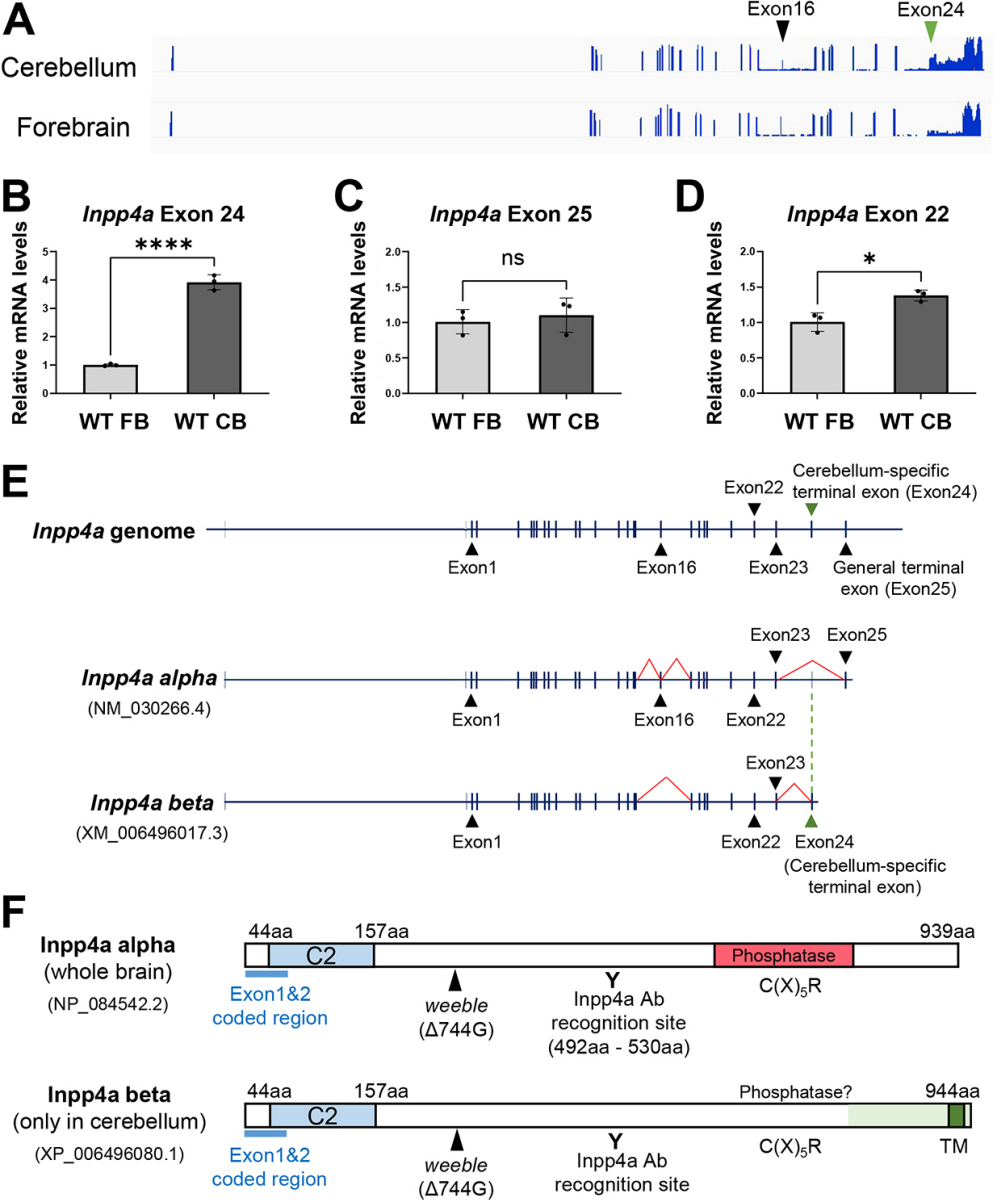
**Identification of cerebellar-specific *Inpp4a* transcripts.** (A) Pile-up view of reads from forebrain and cerebellar RNA-seq data. The black and green arrowheads indicate exon 16 and exon 24, respectively. (B–D) qPCR data of *Inpp4a beta* (B), *Inpp4a alpha* (C) and total *Inpp4a* (D) mRNA in the forebrain (FB) and cerebellum (CB) of WT mice at 2 weeks of age (*n*=3 mice, each brain region). For detection of the transcripts, primers corresponding to exon 25 for *Inpp4a* alpha, exon 24 for *Inpp4a* beta or exon 22 for total *Inpp4a* were used. All values in the graphs are presented as means±s.d. *P<0.05, ****P<0.0001 (unpaired two-tailed Student's *t*-test). ns, not significant. (E) Mouse *Inpp4a* genomic structure and *Inpp4a* transcripts. Black arrowheads indicate the positions of mentioned exons. The green arrowhead indicates cerebellar-specific exon. Red lines indicate positions of distinct splicing. (F) Alignment of amino acid sequences of mouse Inpp4a isoforms (NCBI accessions: NP_084542.2, XP_006496080.1). UniProt provides the 3D structure of Inpp4a protein (E9Q9A0). The blue, red and dark-green boxes indicate the C2 domain, phosphatase domain and putative transmembrane (TM) domain, respectively. The light-green area indicates C-terminus encoded by cerebellar-specific exon. C(X)_5_R is an amino acid sequence essential for phosphatase activity. Black arrowheads indicate the mutation site of *weeble* mutant mice. The resulting frameshift creates a stop codon at amino acid (aa) position 263. ‘Y’ indicates the antibody recognition site of rat monoclonal anti-Inpp4a antibody. Note that part of the phosphatase domain is lacking in the cerebellar isoform of the Inpp4a protein.

The phosphatase domain of *Inpp4a* is encoded by exon 23, and several *Inpp4a* isoforms are generated by alternative splicing or distinct promoter use (Fig. S5) (Shearn et al., 2001). We detected at least two isoforms in the wild-type cerebellum; therefore, we performed RNA sequencing (RNA-seq) on the wild-type forebrain and cerebellum at 2[Supplementary-material sup1]weeks of age. The RNA-seq analysis revealed alternative use of the exon encoding the C-terminal region by the forebrain and cerebellum (Fig. 4A). A previous study reported that alternative inclusion of exon 24 or exon 25 in the C-terminal region of *Inpp4a* generates *Inpp4a beta* and *Inpp4a alpha* isoforms, respectively (Norris et al., 1997). Therefore, we performed qPCR to detect *Inpp4a beta* (exon 24), *Inpp4a alpha* (exon 25) and total *Inpp4a* (exon 22) mRNA (Fig. 4B–D). The *Inpp4a beta* isoform was highly expressed in the cerebellum (Fig. 4B), whereas the *Inpp4a alpha* isoform was expressed at a similar level between forebrain and cerebellum (Fig. 4C). These data are consistent with relatively high expression of total *Inpp4a* transcript in the cerebellum compared with that in the forebrain (Fig. 4D). Thus, the stop codons of the *Inpp4a beta* isoform and the *Inpp4a alpha* isoform are encoded by exon 24 and exon 25, respectively (Fig. 4E). The Inpp4a alpha isoform is expressed in the forebrain and cerebellum; the Inpp4a beta isoform is expressed only in the cerebellum. The Inpp4a beta isoform contains a putative transmembrane domain (Fig. 4F). Furthermore, RNA-seq data showed that exon 16 of *Inpp4a* is often excluded in the cerebellum (Fig. 4A). These data indicate that the cerebellum expresses unique *Inpp4a* isoforms compared with the forebrain. The lower- and higher-molecular mass isoforms in the cerebellum (Fig. 3) seem to correspond to *Inpp4a alpha* and *Inpp4a beta*, respectively.

### Phosphatase activity of N-terminal-truncated Inpp4a

**Fig. 5.**
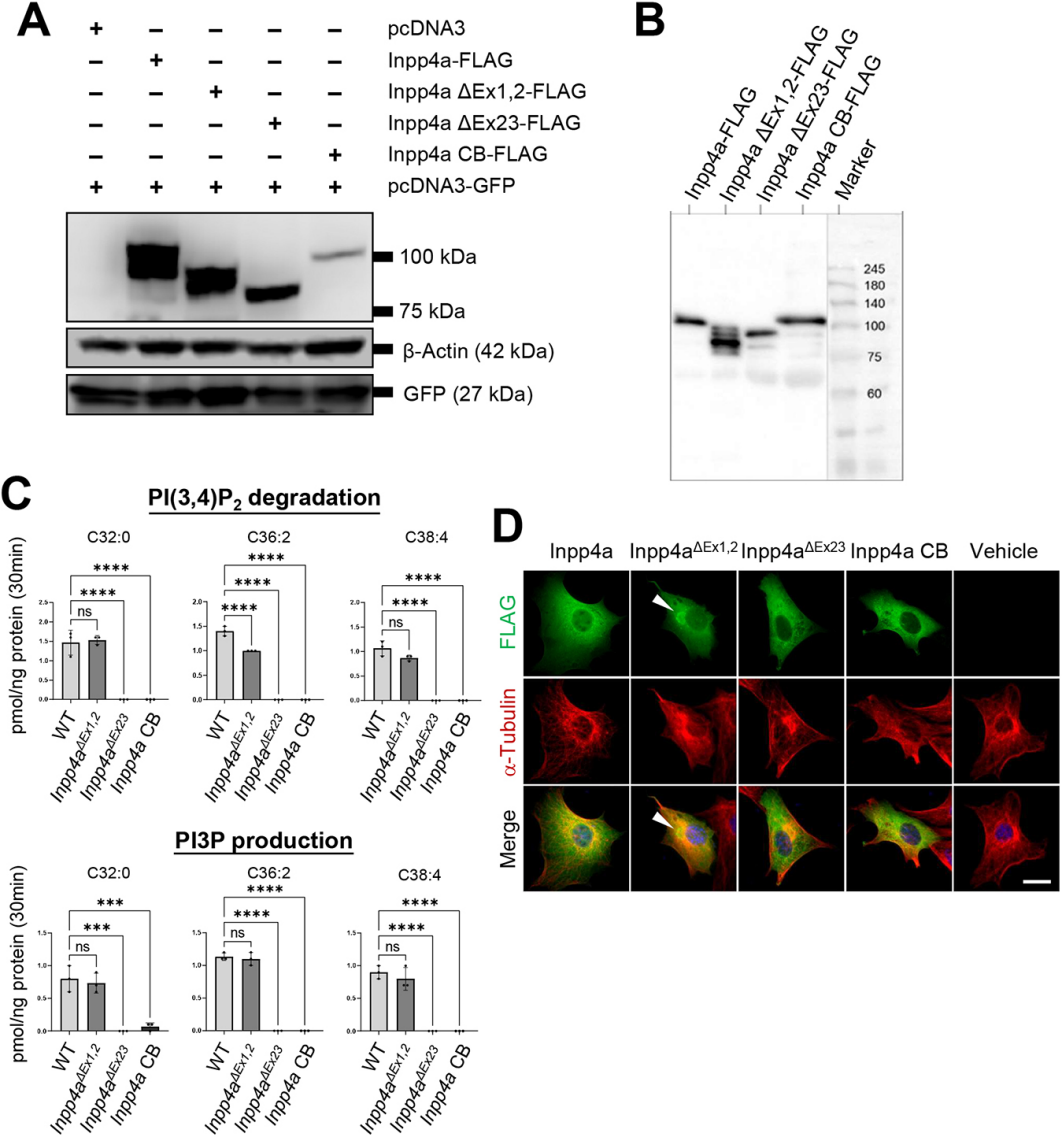
**Expression of Inpp4a isoforms and measurement of phosphatase activity.** (A) Expression of Inpp4a isoforms in HEK293T cells. Inpp4a proteins were detected by western blotting using anti-FLAG antibody (*n*=3 transfections). Lanes are as follows: pcDNA3 and pcDNA3-GFP, pcDNA3-Inpp4a-FLAG and pcDNA3-GFP, pcDNA3-Inpp4a ΔEx1,2-FLAG and pcDNA3-GFP, pcDNA3-Inpp4a ΔEx23-FLAG and pcDNA3-GFP, pcDNA3-Inpp4a CB-FLAG and pcDNA3-GFP. β-Actin was used as an internal control. GFP was used as a control for overexpression. (B) Detection of purified FLAG-tagged Inpp4a proteins by anti-FLAG antibody. Lanes are as follows: pcDNA3-Inpp4a-FLAG, pcDNA3-Inpp4a ΔEx1,2-FLAG, pcDNA3-Inpp4a ΔEx23-FLAG, pcDNA3-Inpp4a CB-FLAG, marker. (C) Measurement of phosphatase activity for PI(3,4)P_2_ of the four FLAG-tagged Inpp4a proteins. Inpp4a-FLAG and Inpp4a ΔEx1,2-FLAG proteins had phosphatase activity, whereas Inpp4a ΔEx23-FLAG protein and Inpp4a CB-FLAG did not. PI3P production was observed for both Inpp4a-FLAG and Inpp4a ΔEx1,2-FLAG proteins. C32:0, 16:0/16:0 PtdIns(3,4)P_2_; C36:2, 18:1/18:1 PtdIns(3,4)P_2_; C38:4, 18:0/20:4 PtdIns(3,4)P_2_. All values in the graphs are presented as means±s.d. ***P<0.001, ****P<0.0001 (one-way ANOVA). ns, not significant. (D) Immunocytochemistry of Inpp4a-FLAG, Inpp4a ΔEx1,2-FLAG, Inpp4a ΔEx23-FLAG and Inpp4a CB-FLAG protein after transfection to NIH3T3 cell line (*n*=2 transfections). Double staining of FLAG-tagged protein (green; top row) and α-tubulin (red; middle row) with DAPI counterstaining (merged; bottom row). Inpp4a ΔEx1,2-FLAG proteins exhibited aggregate-like structures in the cytoplasm (arrowheads). Scale bar: 20 μm.

To investigate the phosphatase activity of the Inpp4a proteins expressed from wild-type, *Inpp4a^ΔEx1,2^* and *Inpp4a^ΔEx23^* alleles towards PtdIns(3,4)P_2_, we constructed a series of plasmids expressing FLAG-tagged Inpp4a proteins (pDNA3-Inpp4a-FLAG, a cerebellar isoform pcDNA3-Inpp4a CB-FLAG, pcDNA3-Inpp4a ΔEx1,2-FLAG and pcDNA3-Inpp4a ΔEx23-FLAG). After transfection of HEK293T cells with these plasmids, we confirmed the production of the proteins by western blotting using an anti-FLAG antibody (Fig. 5A). Notably, the level of Inpp4a CB-FLAG in the transfected cells was lower than that of the other Inpp4a-FLAG proteins. We then assessed the phosphatase activity of purified Inpp4a-FLAG proteins towards PtdIns(3,4)P_2_ (Fig. 5B) by phosphoinositide regioisomer measurement using chiral column chromatography and mass spectrometry (PRMC-MS). Inpp4a has a phosphatase domain located in exon 23; therefore, as expected, Inpp4a ΔEx23-FLAG showed a loss of phosphatase activity compared with the wild type (Fig. 5C). In contrast, Inpp4a ΔEx1,2-FLAG showed only a slight reduction in phosphatase activity (Fig. 5C). Therefore, the retention of phosphatase activity by the N-terminal-truncated protein could be the main reason for the intact cerebellar phenotype in *Inpp4a^ΔEx1,2^* KO mice. Interestingly, a cerebellar Inpp4a isoform (Inpp4a CB-FLAG) had no phosphatase activity towards PtdIns(3,4)P_2_. This result is consistent with a previous report showing the lack of phosphatase activity of the Inpp4a beta isoform expressed in insect cells (Yang et al., 2015). We also examined the intracellular localization of Inpp4a proteins by immunocytochemistry using an anti-FLAG antibody (Fig. 5D). After transfection into NIH3T3 cells, only Inpp4a ΔEx1,2-FLAG showed altered localization in aggregation-like structures, indicating that the N-terminal C2 domain, which binds to PIPs, is essential for proper intracellular localization of Inpp4a.

### Intracellular signaling is altered in *Inpp4a*-deficient cells

**Fig. 6.**
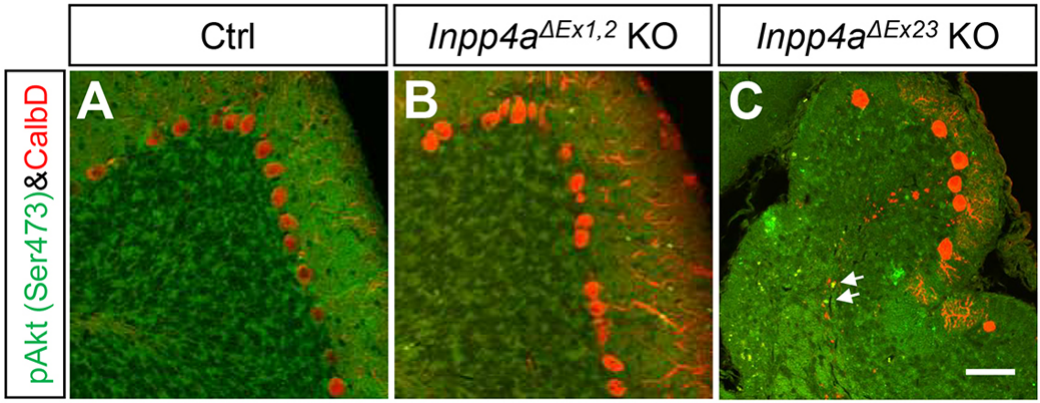
**Activated Akt signaling in the axons of degenerating Purkinje cells.** (A–C) Double IHC for phospho-Akt (pAkt, Ser 473) and calbindin in parasagittal sections of Ctrl (A), *Inpp4a^ΔEx1,2^* KO (B) and *Inpp4a^ΔEx23^* KO (C) mice at 3 weeks of age (*n*=3 mice, each genotype). Strong pAkt dot signals were observed in the axons of Purkinje cells (arrows) in the cerebellar medulla of *Inpp4a^ΔEx23^* KO mice. There were also pAkt-positive cells (green) in the degenerating *Inpp4a^ΔEx23^* KO cerebellum. Scale bar: 100 μm.

*Inpp4a* deficiency results in activation of the Akt pathway through increased levels of PI(3,4)P_2_ (Aich et al., 2012); therefore, we performed IHC in the P21 brain using a well-established phospho-Akt antibody. We detected an upregulated phospho-Akt signal in the degenerating axons of *Inpp4a^ΔEx23^* KO Purkinje cells (Fig. 6C) but not in those of wild-type or *Inpp4a^ΔEx1,2^* KO mice (Fig. 6A,B). The Akt pathway is involved in axon degeneration (Yang et al., 2015); therefore, Akt activation could be involved in the degeneration of Purkinje cell axons in the *Inpp4a^ΔEx23^* KO cerebellum.

### Conditional knockout of *Inpp4a* in the mouse cerebellum

To elucidate the function of Inpp4a in the cerebellum, we generated *En1-Cre*;*Inpp4a* conditional KO (cKO) mice. In *En1-Cre* knock-in mice, Cre recombinase is expressed in cells that express engrailed 1 (En1) during embryonic development in the cerebellum and midbrain (Kimmel et al., 2000), resulting in the deletion of *Inpp4a* from the mesencephalon and rhombomere 1-derived tissues including the cerebellum. To obtain *En1-Cre;Inpp4a* cKO (*En1-Cre;Inpp4a^flox/flox^*) mice, we crossed female *Inpp4a^flox/flox^* mice with male *En1-Cre;Inpp4a^flox/+^* mice.

**Fig. 7.**
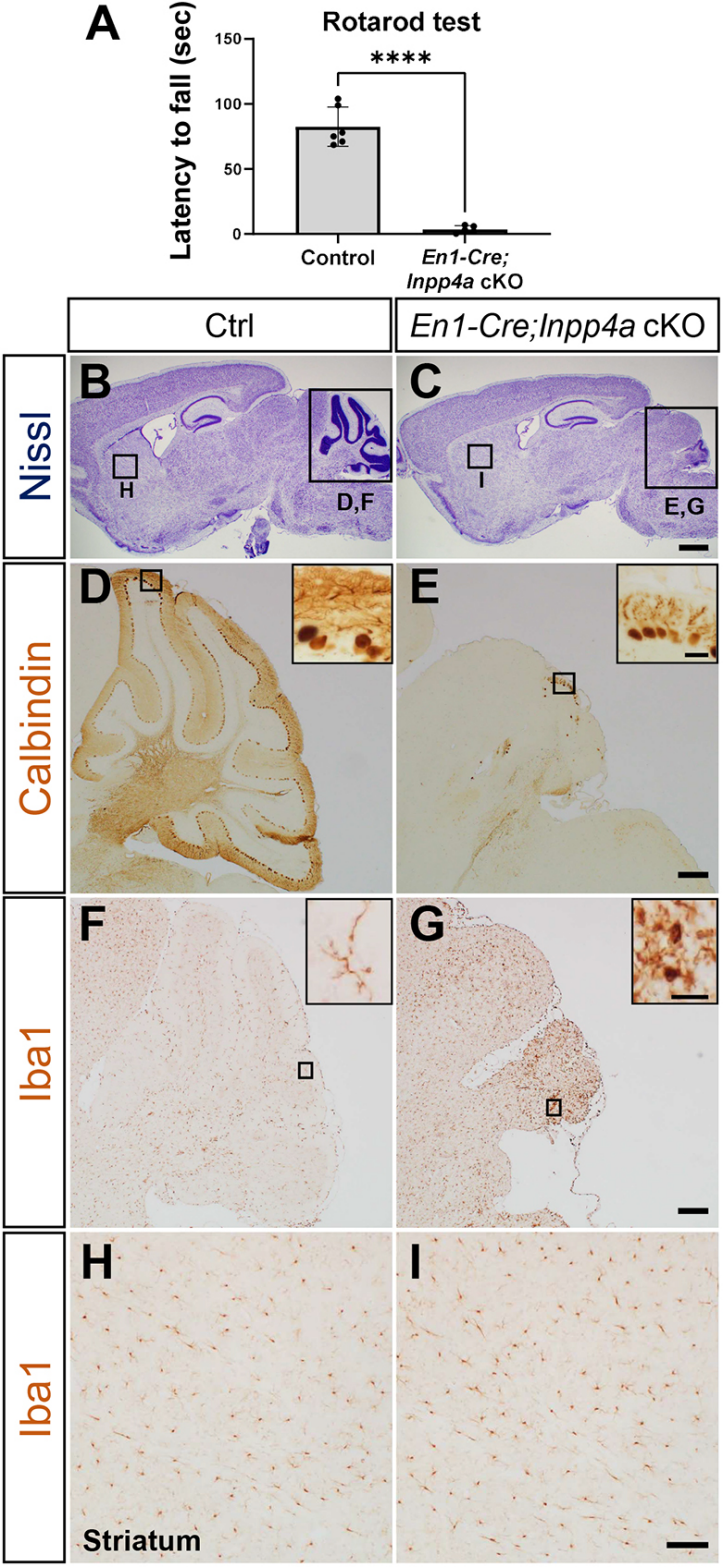
**Cerebellar degeneration in *En1-Cre*;*Inpp4a* cKO mice.** (A) Rotarod test showed less motor coordination in *En1-Cre;Inpp4a* cKO mice compared with Ctrl mice at 3 weeks of age (Ctrl, *n*=6 animals; *Inpp4a* cKO, *n*=5 animals). ****P<0.0001 (unpaired two-tailed Student's *t-*test). (B,C) Nissl staining in parasagittal brain sections of Ctrl (B) and *En1-Cre;Inpp4a* cKO mice (C) at 3 weeks of age (*n*=3 mice, each genotype). *En1-Cre;Inpp4a* cKO mice exhibited cerebellar atrophy. Rectangles indicate the cerebellar (D–G) and striatal (H,I) areas indicated below. (D,E) Calbindin IHC in parasagittal sections of Ctrl (D) and *En1-Cre;Inpp4a* cKO (E) cerebellum at 3 weeks of age (*n*=3 mice, each genotype). Insets show the shape of degenerating Purkinje cells. *En1-Cre;Inpp4a* cKO mice showed cerebellar degeneration. (F,G) Iba1 IHC in parasagittal sections of Ctrl (F) and *En1-Cre;Inpp4a* cKO (G) striatum at 3 weeks of age (*n*=3 mice, each genotype). Insets show the shape of microglia. (H,I) Iba1 IHC in parasagittal sections of Ctrl (H) and *En1-Cre;Inpp4a* cKO (I) striatum at 3 weeks of age. Scale bars: 1 mm (C), 200 μm (E,G), 100 μm (I) and 50 μm (insets).

*En1-Cre*;*Inpp4a* cKO mice exhibited ataxia and a lifespan of only 4–5 weeks (Fig. 7A; Movie 4). Histological analyses showed severe atrophy of the cerebellum but not of other brain regions at the age of 3 weeks (Fig. 7B,C). In addition, calbindin and Iba1 (also known as Aif1) IHC indicated apparent Purkinje cell loss (Fig. 7D,E) and activated microglia (Fig. 7F,G) in the cerebellum, respectively. In contrast, Iba1 IHC in the striatum was similar between control and *En1-Cre*;*Inpp4a* cKO mice, indicating an intact striatum (Fig. 7H,I). These behavioral and histological defects are similar to those of *Inpp4a^ΔEx23^* KO mice, indicating that disruption of *Inpp4a* in the cerebellum is the leading cause of cerebellar atrophy and movement disorder in *Inpp4a^ΔEx23^* KO mice.

## DISCUSSION

**Fig. 8.**
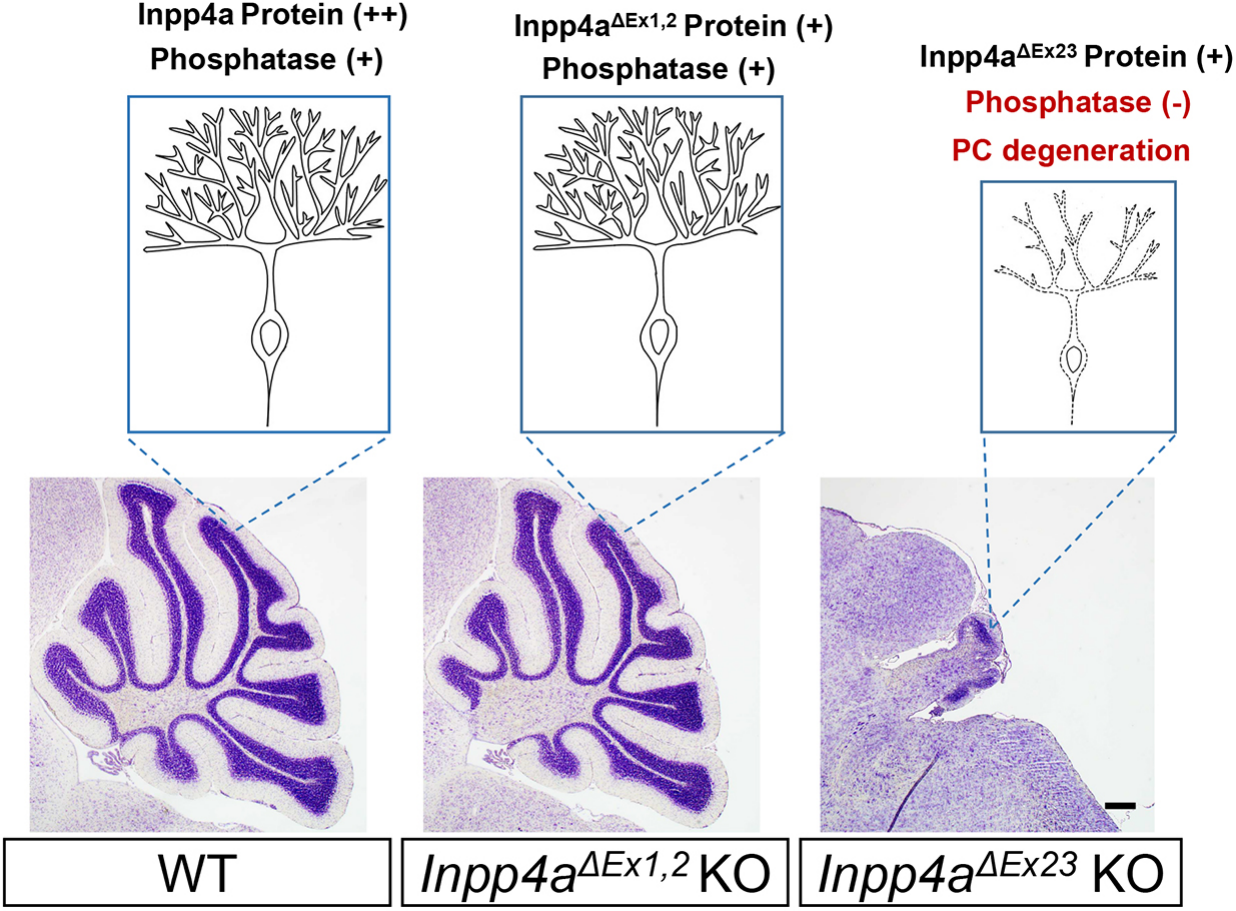
**Schematic diagrams of Purkinje cell phenotype.** Schematic diagrams of the Purkinje cell phenotype in WT, *Inpp4a^ΔEx1,2^* KO and *Inpp4a^ΔEx23^* KO mice. Nissl staining was performed on 3-week-old sections. Scale bar: 200 μm.

Here, we investigated two lines of *Inpp4a* mutant mice side by side, and we report their phenotypic diversity. Specifically, although striatal degeneration occurred in both *Inpp4a^ΔEx23^* and *Inpp4a^ΔEx1,2^* mutant lines, cerebellar degeneration was observed only in *Inpp4a^ΔEx23^* KO mice. Conversely, pain-induced epilepsy was observed only in *Inpp4a^ΔEx1,2^* KO mice. This is the first report on the attenuated cerebellar phenotypes and pain-induced epilepsy in *Inpp4a^ΔEx1,2^* KO mice. Furthermore, we analyzed the properties of the *Inpp4a* transcripts and proteins from each mutant allele regarding exon composition and phosphatase activity. We found the expression of a previously unknown N-terminal-truncated Inpp4a isoform in the cerebellum of *Inpp4a^ΔEx1,2^* KO mice. This isoform was produced by alternative translation initiation and retained phosphatase activity toward PI(3,4)P_2_. In contrast, the *Inpp4a^ΔEx23^*-encoded protein had no enzymatic activity. These data indicate that the N-terminal-truncated Inpp4a with phosphatase activity is responsible for the attenuated cerebellar phenotype in the cerebellum of *Inpp4a^ΔEx1,2^* KO mice (Fig. 8). These differences in phosphatase activities possibly explain the phenotypic diversity in the cerebellum caused by different *Inpp4a* mutations. In addition, *Inpp4a* mutant mice are good models of human diseases caused by *INPP4A* mutations, which also exhibit symptomatic diversity. There are registrations of nonsense mutations within many *INPP4A* exons in the dbSNP database, which seem to be pathogenic (Table S1). It is possible that there are unreported novel human *INPP4A* mutant diseases.

Western blotting indicated two Inpp4a isoforms in the wild-type cerebellum. A comparison of RNA-seq data between wild-type forebrain and cerebellum revealed an alternative exon in the cerebellar transcripts. This cerebellar exon encodes a putative transmembrane domain and is known as INPP4 type I beta (Norris et al., 1997). The isoform had no phosphatase activity when expressed in bacteria or insect SF9 cells (Norris et al., 1997). In this study, transfection in mammalian HEK293T cells revealed that the wild-type Inpp4a isoform has phosphatase activity toward PIP_2_, whereas the cerebellar Inpp4a (Inpp4a beta) isoform does not. Because homozygous mutation in the cerebellar INPP4A isoform leads to neurological disorder without central nervous system malformation (Najmabadi et al., 2011), the isoform with the C2 domain and putative transmembrane domain has specific function(s) other than phosphatase activity. One possible function is synaptic modulation (Cremona and de Camilli, 2001; Sasaki et al., 2010) because synaptotagmin proteins (Chapman, 2002) also have C2 and transmembrane domains. The second possibility is a nuclear function, such as the stress response (Gozani et al., 2003) or DNA repair, similar to INPP4B (Sun et al., 2020). Notably, there is also an INPP4B isoform with a putative transmembrane domain at the C-terminus (Norris et al., 1997).

In the *Inpp4a^ΔEx1,2^* brain, we observed neuronal cell degeneration in the striatum but not in cerebellar Purkinje cells. A possible reason for this is that the high promoter activity of *Inpp4a* in Purkinje cells allows *Inpp4a* mRNA to be present in large quantities, allowing sufficient truncated Inpp4a protein to be produced despite less effective alternative translation initiation. Another possibility is that striatal neurons are more sensitive to reduced levels of Inpp4a and phosphatase activity; for example, less Inpp4b in the striatum leads to less redundancy. Alternatively, the function of the N-terminal C2 domain may be essential for the survival of striatal neurons. Alternative translation initiation occurs in many other genes (Gurvich et al., 2009; Lock et al., 1991; Xu and Zhang, 2020), and, in the generation of knockout mice, knocking out the exon encoding the first ATG sometimes results in a hypomorphic instead of a null allele that can result in a weak phenotype (Motley et al., 2020; Zhou et al., 2022). Recent studies have reported human genetic diseases associated with variation in the human *INPP4A* gene. These diseases have diverse manifestations with or without cerebellar symptoms (Banihashemi et al., 2020; Hecher et al., 2023; Najmabadi et al., 2011; Özkan Kart et al., 2023; Sheffer et al., 2015). This diversity could be caused by the location of the *INPP4A* mutations. It is possible that the N-terminal-truncated INPP4A protein is produced from an *INPP4A* mutant allele, such as p.(Gln39*) mutation (Banihashemi et al., 2020). Indeed, *Inpp4a^ΔEx1,2^* transcripts isolated by reverse transcription PCR from *Inpp4a^ΔEx1,2^* KO mice have a sequence in exon 3 that corresponds to a Kozak sequence (Kozak, 1977). It is also possible that translation initiation can start from a non-ATG codon (Florkiewicz and Sommer, 1989).

The N-terminal-truncated Inpp4a protein expressed from the *Inpp4a^ΔEx1,2^* allele formed aggregate-like structures in the cytoplasm when we examined subcellular localization in transfected cells. Notably, Inpp4a proteins contain multiple Atg8-interacting (WxxL) motifs (Noda et al., 2010), and some of the motifs may have a functional interaction with Atg8, an autophagy-related protein required for autophagosome formation. Furthermore, the N-terminal-truncated Inpp4a protein lacking the C2 domain may result in abnormal intracellular localization of the protein and influence the regulation of autophagy, which could explain the aggregation-like structure after its overexpression.

In the present study, we analyzed two strains of *Inpp4a* mutant mice with or without cerebellar degeneration. We found low levels of N-terminal-truncated Inpp4a via alternative translation initiation in *Inpp4a^ΔEx1,2^* KO mice with phosphatase activity for PIP_2_. In contrast, the mutant Inpp4a protein expressed from the *Inpp4a^ΔEx23^* allele did not exhibit phosphatase activity for PIP_2_. The phosphatase activity may account for the attenuated cerebellar phenotypes in the *Inpp4a^ΔEx1,2^* KO mice. These *Inpp4a* mutant mouse strains are good models for developing new treatment strategies for human INPP4A disease, which has diverse symptoms.

## MATERIALS AND METHODS

### Animals

We transferred two *Inpp4a* mutant lines of C57BL/6J background from Tokyo Medical and Dental University to Niigata University: *Inpp4a^tm1Tsak^* mice (MGI:4462378) (Sasaki et al., 2010) and *Inpp4a^flox^* mice (Nigorikawa et al., 2015). In this study, *Inpp4a^tm1Tsak^* allele is also called *Inpp4a^ΔEx1,2^*, which lacks part of the N-terminal C2 domain. In the *Inpp4a^flox^* allele, two loxP sites are located upstream and downstream of exon 23, encoding part of the phosphatase domain (Peters et al., 1998). To generate *Inpp4a^ΔEx23^* allele, we crossed *Inpp4a^flox^* mice with β-actin (*Actb)-iCre-IRES-GFP* knock-in mice of C57BL/6N background (Zhou et al., 2018). Double heterozygous mice (*Actb-iCre-IRES-GFP*;*Inpp4a^flox^*^/+^) were crossed with C57BL/6N mice to generate the *Inpp4a* KO allele lacking exon 23 (*Inpp4a^ΔEx23^*). *Inpp4a* KO mice (*Inpp4a^ΔEx1,2/ΔEx1,2^* or *Inpp4a^ΔEx23/ΔEx23^*) were generated by crossing heterozygous pairs. For cKO experiments, we used *En1^tm2(Cre)Wrst^* (MGI:2446434; Kimmel et al., 2000). We crossed female *Inpp4a^flox/flox^* mice with male *En1-Cre;Inpp4a^flox/+^* mice to obtain *En1-Cre;Inpp4a* cKO (*En1-Cre;Inpp4a^flox/flox^*) mice. The mice were maintained at 22±2°C and 60% humidity on a 12 h light/dark cycle. Food and water were freely accessible. Animal care and experimental protocols were approved by the Animal Experiment Committee of Niigata University and Tokyo Medical and Dental University. We used male and female mice at 1–3 weeks of age.

### Genotyping PCR

Genotyping PCR for the *Inpp4a^ΔEx1,2^* allele was performed as previously described (MGI:4462378; Sasaki et al., 2010). Primers used to detect *Inpp4a^flox^* and *Inpp4a^+^* alleles were previously described (Morioka et al., 2022). *Inpp4a^ΔEx23^* allele was detected using the following primers (5′-AGGGTCAGTGTGAAGCAGTGATG-3′ and 5′-TGTCGCCACTTTTGCTCCTAT C-3′), which produce 510 bp product from *Inpp4a^ΔEx23^* allele and 1350 bp product from wild-type allele. For genotyping of *Actb-iCre-IRES-GFP* knock-in mice (Zhou et al., 2018), *iCre* 538 primers (*iCre* 538-F, 5′-CTCAACATGCTGCACAGGAGAT-3′; *iCre* 538-R, 5′-ACCATAGATCAGGCGGTGGGT-3′) were used to amplify 538 bp fragments from the *iCre* transgene. PCR was performed using Quick Taq HS Dye Mix (Toyobo, Osaka, Japan) and PCR Cyclers (TaKaRa Bio, Shiga, Japan) under the following PCR conditions: 95°C for 30 s, 30 cycles of 95°C for 10 s, 60°C for 30 s and 72°C for 30 s, followed by 72°C for 60 s. The PCR products were separated by electrophoresis on 2% agarose gel.

### Histology

Paraffin sections for IHC and ISH were prepared as previously described (Takebayashi et al., 2000; Yoshioka et al., 2020). Consecutive 10 μm-thick coronal and sagittal sections of brains were cut using a rotary paraffin microtome (HM 325, Microm, Walldorf, Germany). The following primary antibodies were used: mouse anti-calbindin-D (1:10,000; 300, Swant, Burgdorf, Switzerland), rabbit anti-cleaved caspase-3 (1:400; 9664, Cell Signaling Technology), rabbit anti-ssDNA (1:100; 18731, IBL), rabbit anti-GFAP (1:100; 442251, Nichirei, Tokyo, Japan), rat anti-Inpp4a [1:500; homemade, immunogen is 492–530 amino acids (aa) of mouse Inpp4a], rabbit anti-Iba1 (1:2000; 019-19741, WAKO) and rabbit phospho-Akt (1:100; 4060, Cell Signaling Technology). In addition, peroxidase-conjugated secondary antibodies were used, including goat anti-rabbit IgG (1:200; 458, MBL, Nagoya, Japan), goat anti-mouse IgG (1:200; 330, MBL), rabbit anti-goat IgG (1:200; 546, MBL) or rabbit anti-rat IgG (1:200; P0450, Dako). After 3,3′-diaminobenzidine staining, sections were mounted with coverslips. For fluorescent IHC, the following secondary antibodies were used: Alexa Fluor488-conjugated goat anti-rabbit IgG (1:1000; A11034, Invitrogen/Thermo Fisher Scientific) and Alexa Fluor594-conjugated goat anti-mouse IgG (1:1000; A11032, Invitrogen/Thermo Fisher Scientific). Quantification of ssDNA, cl-Casp3, Iba1 and GFAP was performed by MetaMorph software (Meta Series Software ver. 7.10.2, Molecular Devices, San Jose, CA, USA). The number of positive signals was normalized by the area of the cerebellum (ssDNA, cl-Casp3) or the striatum (Iba1, GFAP) in each section. Quantification analysis was performed on three sections per mouse, with three or more mice per group.

As previously described, ISH was performed on paraffin sections (Horie et al., 2014; Takebayashi et al., 2000) using a mouse *Inpp4a* probe [GenBank accession number: NM_030266, nucleotides 927–1825]. After ISH, sections were counterstained by Nuclear Fast Red. Light microscopic images were taken using a microscope (BX53, Olympus) connected to a CCD camera (DP74, Olympus) or a confocal microscopy FV-1200 (Olympus).

### RNA-seq analyses

RNA-seq was performed according to previous reports (Bizen et al., 2022; Yoshioka et al., 2022). RNA was extracted from the wild-type forebrain after removing the olfactory bulb and wild-type cerebellum at P14 using an RNeasy Mini Kit (Qiagen).

### Real-time PCR

Total RNA was extracted from the mouse brain using an RNeasy Mini Kit (Qiagen), including DNase digestion; 100 ng of RNA template was used for cDNA synthesis with oligonucleotide (dT) primers. Real-time PCR was performed using a StepOnePlus Real-Time PCR system (Thermo Fisher Scientific) and the following cycling conditions: 95°C for 2 min, followed by 40 cycles of 95°C for 15 s and 60°C for 40 s, and then 95°C for 15 s, 60°C for 1 min and 95°C for 15 s. Gene expression levels were analyzed using the ΔΔCT method. *Gapdh* was used as an internal control for normalization. The primers used for real-time PCR are as follows: *Inpp4a* forward, 5′-ACTCCATCGCTAGATCGAAAACC-3′; *Inpp4a* reverse, 5′-AGGCAATGCTGCTTAGAAAGAT-3′ (for *Inpp4a* in Fig. 3); exon 22 forward, 5′-TCTACCTCGATCTCGGAGTCA-3′; exon 22 reverse, 5′-TGCGTGCATGGACATTCTGT-3′ (for total *Inpp4a*); exon 24 forward, 5′-ACCCAGAAGAACTTGAGCGG-3′; exon 24 reverse, 5′-CACCAGGTACGCTATGCTCA-3′ (for *Inpp4a beta*); exon 25 forward, 5′-GTTGTCGGCGAGAAAACACA-3′; exon 25 reverse, 5′-CGTAAGTCCCTTCTGGAGGC-3′ (for *Inpp4a alpha*); *Gapdh* forward, 5′-AGGTCGGTGTGAACGGATTTG-3′; *Gapdh* reverse, 5′-TGTAGACCATGTAGTTGAGGTCA-3′ (for internal control).

### Construction of mouse *Inpp4a* expression plasmids

Mouse *Inpp4a* cDNAs with C-terminal FLAG tag were generated by PCR using mouse cerebellum cDNAs (wild-type, *Inpp4a^ΔEx1,2^* KO and *Inpp4a^ΔEx23^* KO mice) as templates. The primers were 5′-GGGGTACCCCCCACGTGGTCCAAAAGCAAG-3′ (sense), 5′-ATAAGAATGCGGCCGCAAGCTTTCACTTGTCATCGTCATCCTTGTAGTCTGTCTCAACTTTTCCGTAAGTCCCT-3′ (antisense for Inpp4a WT, Δ2, Δ23) and 5′-ATAAGAATGCGGCCGCAAGCTTTCACTTGTCATCGTCATCCTTGTAGTCCGGGCACTTTTGTCTGCCTC-3′ (antisense for Inpp4a CB). TaKaRa LA Taq (TaKaRa Bio.) was used for the PCR reactions. The PCR products containing the full-length *Inpp4a* cDNAs with FLAG tag were cut with Asp718 (Roche) and NotI (Nippon Gene) and then subcloned into pcDNA3 plasmid vector (Invitrogen). The produced plasmids are referred to as pcDNA3-mouse Inpp4a-FLAG, pcDNA3-mouse Inpp4a ΔEx1,2-FLAG, pcDNA3-mouse Inpp4a ΔEx23, and pcDNA3-mouse Inpp4a CB-FLAG plasmids. Sequencing was performed on both strands.

### Cell culture, transfection and immunocytochemistry

Cell culture, transfection and immunocytochemistry were performed as previously described with minor modifications (Bizen et al., 2022). NIH3T3 cells (Thumkeo et al., 2011) were plated on coverslips pre-coated with poly-L-ornithine (Sigma-Aldrich) and fibronectin (Thermo Fisher Scientific) and cultured in Dulbecco's modified Eagle medium with 10% fetal bovine serum. The next day, pcDNA3-mouse Inpp4a-FLAG, pcDNA3-mouse Inpp4a ΔEx1,2-FLAG, pcDNA3-mouse Inpp4a ΔEx23, and pcDNA3-mouse Inpp4a CB-FLAG plasmids were transfected into NIH3T3 cells using Lipofectamine 3000 (Thermo Fisher Scientific) according to the manufacturer's protocol. Forty-eight hours after transfection, the cells were fixed with 4% paraformaldehyde for 15 min. After washing three times with PBS, the cells were incubated for 1 h with PBST (PBS and 0.1% Triton X-100) containing 10% goat serum for blocking and permeabilization. The cells were further incubated with rabbit polyclonal anti-FLAG antibody (1:1000; M185-3 L, MBL) and anti-α-tubulin antibody (1:1000; 3873, Cell Signaling Technology) in antibody solution (PBST and 10% goat serum) overnight at 4°C. The following day, the cells were washed three times with PBST and then incubated with an antibody solution containing secondary antibodies for 1 h at room temperature. After washing three times with PBST, the cells were incubated with 4′,6-diamino-2-phenylindole (DAPI; Dojindo, Kumamoto, Japan) for counterstaining and washed three times with PBS. The images were collected using an Olympus microscope (BX53, Olympus) and a digital camera system (DP74, Olympus).

### Western blotting

Protein lysates from central nervous system tissues and cultured cells were prepared as previously described (Bizen et al., 2022; Yoshioka et al., 2022). After transfection to HEK293T cells using polyethylenimine (PEI) Max (Polysciences, purchased from Cosmo Bio) according to the manufacturer's protocol, western blotting was performed as previously described (Bizen et al., 2022). The following antibodies were used: rat monoclonal anti-Inpp4a (1:2000; homemade), mouse monoclonal anti-FLAG (1:1000; F1804, Sigma-Aldrich), rabbit anti-GFP (1:20,000; MBL) and mouse monoclonal anti-β-actin (1:20,000; A5441, Sigma-Aldrich).

### Measurement of phosphatase activity of Inpp4a proteins

FLAG-tagged Inpp4a, Inpp4a ΔEx1,2, Inpp4a ΔEx23 or Inpp4a CB was expressed in HEK293T cells and purified using an anti-FLAG antibody (Sigma-Aldrich) as previously described (Kofuji et al., 2015). Recombinant Inpp4a-FLAG, Inpp4a ΔEx1,2-FLAG, Inpp4a ΔEx23-FLAG or Inpp4a CB-FLAG (50 ng) was incubated for 30 min at 37°C with 5–10 µmol/l 16:0/16:0 PtdIns(3,4)P_2_ (Cayman Chemical), 18:1/18:1 PtdIns(3,4)P_2_ (Avanti Polar Lipids) and 18:0/20:4 PtdIns(3,4)P_2_ (Avanti Polar Lipids) in 25 mmol/l HEPES (pH7.4) plus 100 mmol/l NaCl and 2 mmol/l dithiothreitol. Each enzyme heat treated at 80°C for 10 min was used as a control. Degradation of PtdIns(3,4)P_2_ and production of PdInsP were detected by liquid chromatography–tandem mass spectrometry (LC-MS/MS) (Koizumi et al., 2019). The production of PdIns(3)P was determined by phosphoinositide regioisomer measurement by chiral column chromatography and mass spectrometry (Morioka et al., 2022). Briefly, the reaction mixture was transferred to a glass tube and mixed with 700 μl methanol/chloroform (1/1) containing 1 nmol 8:0/8:0 PI(4,5)P_2_ (as an absorption inhibitor, Cayman Chemical) and 10 pmol each of synthetic C17:0/C20:4 phosphoinositides (Avanti Polar Lipids) as internal standards, followed by a methylation reaction using 75 μl trimethylsilyl diazomethane (Tokyo Chemical Industry) for 5 min at room temperature. After the reaction was quenched with 7.5 μl glacial acetic acid, the sample was mixed with 700 μl methanol/chloroform (1/1), followed by vortexing for 1 min. After centrifugation at 1200 ***g*** for 3 min, the lower phase was taken to dry under a stream of nitrogen, then re-dissolved in 100 μl methanol (for the C18 column) or acetonitrile (for the chiral column). The level of methylated phosphoinositides was measured by LC-MS/MS under the same conditions as described (Koizumi et al., 2019; Morioka et al., 2022).

### Rotarod test

The rotarod test was performed as previously described (Yoshioka et al., 2022). The latency to fall from a rotating rod (30 mm diameter) with an acceleration from 10 to 150 rpm was measured. Each trial was performed for 3 min. For each mouse, two trials were conducted in a day.

### Statistical analysis

Datasets from two groups or more were analyzed by unpaired two-tailed Student's *t*-test or one-way ANOVA with appropriate post hoc test. All datasets were tested for normal distribution. If data were not normally distributed, appropriate nonparametric tests were performed. Statistical analysis was performed using ANOVA4 on the Web. Data are presented as the mean±s.d. *P*<0.05 was considered significant. *P*-values and statistical tests used are indicated in the figure legends. No statistical methods were used to predetermine sample sizes. The sample size was determined empirically using criteria commonly employed in the field. No data were excluded from analyses.

### Study approval

All experimental protocols were conducted following the guidelines for animal care regulated by the animal committee of Niigata University and Tokyo Medical Dental University, Japan.

## Supplementary Material

10.1242/dmm.050169_sup1 Supplementary information

## References

[DMM050169C1] Aich, J., Mabalirajan, U., Ahmad, T., Agrawal, A. and Ghosh, B. (2012). Loss-of-function of inositol polyphosphate-4-phosphatase reversibly increases the severity of allergic airway inflammation. *Nat. Commun.* 3, 877. 10.1038/ncomms1880 22673904

[DMM050169C2] Balla, T. (2013). Phosphoinositides: tiny lipids with giant impact on cell regulation. *Physiol. Rev.* 93, 1019-1137. 10.1152/physrev.00028.2012 23899561 PMC3962547

[DMM050169C3] Banihashemi, S., Tahmasebi-Birgani, M., Mohammadiasl, J. and Hajjari, M. (2020). Whole exome sequencing identified a novel nonsense INPP4A mutation in a family with intellectual disability. *Eur. J. Med. Genet.* 63, 103846. 10.1016/j.ejmg.2020.103846 31978615

[DMM050169C4] Bizen, N., Bepari, A. K., Zhou, L., Abe, M., Sakimura, K., Ono, K. and Takebayashi, H. (2022). Ddx20, an Olig2 binding factor, governs the survival of neural and oligodendrocyte progenitor cells via proper Mdm2 splicing and p53 suppression. *Cell Death Differ.* 29, 1028-1041. 10.1038/s41418-021-00915-8 34974536 PMC9090832

[DMM050169C5] Bunney, T. D. and Katan, M. (2010). Phosphoinositide signalling in cancer: beyond PI3K and PTEN. *Nat. Rev. Cancer* 10, 342-352. 10.1038/nrc2842 20414202

[DMM050169C6] Chapman, E. R. (2002). Synaptotagmin: a Ca^2+^ sensor that triggers exocytosis? *Nat. Rev. Mol. Cell Biol.* 3, 498-508. 10.1038/nrm855 12094216

[DMM050169C7] Cremona, O. and De Camilli, P. (2001). Phosphoinositides in membrane traffic at the synapse. *J. Cell Sci.* 114, 1041-1052. 10.1242/jcs.114.6.1041 11228149

[DMM050169C8] Echard, A. (2012). Phosphoinositides and cytokinesis: the “PIP” of the iceberg. *Cytoskeleton* 69, 893-912. 10.1002/cm.21067 23012232

[DMM050169C9] Florkiewicz, R. Z. and Sommer, A. (1989). Human basic fibroblast growth factor gene encodes four polypeptides: three initiate translation from non-AUG codons. *Proc. Natl. Acad. Sci. USA* 86, 3978-3981. 10.1073/pnas.86.11.3978 2726761 PMC287371

[DMM050169C10] Fukumoto, M., Ijuin, T. and Takenawa, T. (2017). PI(3,4)P_2_ plays critical roles in the regulation of focal adhesion dynamics of MDA-MB-231 breast cancer cells. *Cancer Sci.* 108, 941-951. 10.1111/cas.13215 28247964 PMC5448597

[DMM050169C11] Gozani, O., Karuman, P., Jones, D. R., Ivanov, D., Cha, J., Lugovskoy, A. A., Baird, C. L., Zhu, H., Field, S. J., Lessnick, S. L. et al. (2003). The PHD finger of the chromatin-associated protein ING2 functions as a nuclear phosphoinositide receptor. *Cell* 114, 99-111. 10.1016/S0092-8674(03)00480-X 12859901

[DMM050169C12] Gurvich, O. L., Maiti, B., Weiss, R. B., Aggarwal, G., Howard, M. T. and Flanigan, K. M. (2009). *DMD* exon 1 truncating point mutations: amelioration of phenotype by alternative translation initiation in exon 6. *Hum. Mutat.* 30, 633-640. 10.1002/humu.20913 19206170 PMC2663021

[DMM050169C13] Hawkins, P. T. and Stephens, L. R. (2016). Emerging evidence of signalling roles for PI(3,4)*P*_2_ in Class I and II PI3K-regulated pathways. *Biochem. Soc. Trans.* 44, 307-314. 10.1042/BST20150248 26862220

[DMM050169C14] Hecher, L., Harms, F. L., Lisfeld, J., Alawi, M., Denecke, J. and Kutsche, K. (2023). INPP4A-related genetic and phenotypic spectrum and functional relevance of subcellular targeting of INPP4A isoforms. *Neurogenetics* 24, 79-93. 10.1007/s10048-023-00709-9 36653678

[DMM050169C15] Horie, M., Watanabe, K., Bepari, A. K., Nashimoto, J., Araki, K., Sano, H., Chiken, S., Nambu, A., Ono, K., Ikenaka, K. et al. (2014). Disruption of actin-binding domain-containing Dystonin protein causes *dystonia musculorum* in mice. *Eur. J. Neurosci.* 40, 3458-3471. 10.1111/ejn.12711 25195653

[DMM050169C16] Kim, J.-S., Yun, H. S., Um, H.-D., Park, J. K., Lee, K.-H., Kang, C.-M., Lee, S.-J. and Hwang, S.-G. (2012). Identification of inositol polyphosphate 4-phosphatase type II as a novel tumor resistance biomarker in human laryngeal cancer HEp-2 cells. *Cancer Biol. Ther.* 13, 1307-1318. 10.4161/cbt.21788 22895072 PMC3493439

[DMM050169C17] Kimmel, R. A., Turnbull, D. H., Blanquet, V., Wurst, W., Loomis, C. A. and Joyner, A. L. (2000). Two lineage boundaries coordinate vertebrate apical ectodermal ridge formation. *Genes Dev.* 14, 1377-1389. 10.1101/gad.14.11.1377 10837030 PMC316660

[DMM050169C18] Kofuji, S., Kimura, H., Nakanishi, H., Nanjo, H., Takasuga, S., Liu, H., Eguchi, S., Nakamura, R., Itoh, R., Ueno, N. et al. (2015). INPP4B is a PtdIns(3,4,5)P_3_ phosphatase that can act as a tumor suppressor. *Cancer Discov.* 5, 730-739. 10.1158/2159-8290.CD-14-1329 25883023

[DMM050169C19] Koizumi, A., Narita, S., Nakanishi, H., Ishikawa, M., Eguchi, S., Kimura, H., Takasuga, S., Huang, M., Inoue, T., Sasaki, J. et al. (2019). Increased fatty acyl saturation of phosphatidylinositol phosphates in prostate cancer progression. *Sci. Rep.* 9, 13257. 10.1038/s41598-019-49744-3 31520002 PMC6744559

[DMM050169C20] Kozak, M. (1977). Nucleotide sequences of 5′-terminal ribosome-protected initiation regions from two reovirus messages. *Nature* 269, 390-394. 10.1038/269390a0 909586

[DMM050169C21] Lock, P., Ralph, S., Stanley, E., Boulet, I., Ramsay, R. and Dunn, A. R. (1991). Two isoforms of murine hck, generated by utilization of alternative translational initiation codons, exhibit different patterns of subcellular localization. *Mol. Cell. Biol.* 11, 4363-4370. 10.1128/mcb.11.9.4363-4370.1991 1875927 PMC361298

[DMM050169C22] Morioka, S., Nakanishi, H., Yamamoto, T., Hasegawa, J., Tokuda, E., Hikita, T., Sakihara, T., Kugii, Y., Oneyama, C., Yamazaki, M. et al. (2022). A mass spectrometric method for in-depth profiling of phosphoinositide regioisomers and their disease-associated regulation. *Nat. Commun.* 13, 83. 10.1038/s41467-021-27648-z 35013169 PMC8749000

[DMM050169C23] Motley, W. W., Züchner, S. and Scherer, S. S. (2020). Isoform-specific loss of dystonin causes hereditary motor and sensory neuropathy. *Neurol. Genet* 6, e496. 10.1212/NXG.0000000000000496 32802955 PMC7413632

[DMM050169C24] Najmabadi, H., Hu, H., Garshasbi, M., Zemojtel, T., Abedini, S. S., Chen, W., Hosseini, M., Behjati, F., Haas, S., Jamali, P. et al. (2011). Deep sequencing reveals 50 novel genes for recessive cognitive disorders. *Nature* 478, 57-63. 10.1038/nature10423 21937992

[DMM050169C25] Nigorikawa, K., Hazeki, K., Sasaki, J., Omori, Y., Miyake, M., Morioka, S., Guo, Y., Sasaki, T. and Hazeki, O. (2015). Inositol polyphosphate-4-phosphatase type I negatively regulates phagocytosis via dephosphorylation of phagosomal PtdIns(3,4)P2. *PLoS One* 10, e0142091. 10.1371/journal.pone.0142091 26535897 PMC4633150

[DMM050169C26] Nilius, B., Owsianik, G. and Voets, T. (2008). Transient receptor potential channels meet phosphoinositides. *EMBO J.* 27, 2809-2816. 10.1038/emboj.2008.217 18923420 PMC2570475

[DMM050169C27] Noda, N. N., Ohsumi, Y. and Inagaki, F. (2010). Atg8-family interacting motif crucial for selective autophagy. *FEBS Lett.* 584, 1379-1385. 10.1016/j.febslet.2010.01.018 20083108

[DMM050169C28] Norris, F. A., Auethavekiat, V. and Majerus, P. W. (1995). The isolation and characterization of cDNA encoding human and rat brain inositol polyphosphate 4-phosphatase. *J. Biol. Chem.* 270, 16128-16133. 10.1074/jbc.270.27.16128 7608176

[DMM050169C29] Norris, F. A., Atkins, R. C. and Majerus, P. W. (1997). The cDNA cloning and characterization of inositol polyphosphate 4-phosphatase type II. *J. Biol. Chem.* 272, 23859-23864. 10.1074/jbc.272.38.23859 9295334

[DMM050169C30] Nystuen, A., Legare, M. E., Shultz, L. D. and Frankel, W. N. (2001). A null mutation in inositol polyphosphate 4-phosphatase type I causes selective neuronal loss in weeble mutant mice. *Neuron* 32, 203-212. 10.1016/S0896-6273(01)00468-8 11683991

[DMM050169C31] Özkan Kart, P., Citli, S., Yildiz, N. and Cansu, A. (2023). A novel INPP4A mutation with pontocerebellar hypoplasia, myoclonic epilepsy, microcephaly, and severe developmental delay. *Brain Dev.* 45, 300-305. 10.1016/j.braindev.2023.01.006 36759255

[DMM050169C32] Peters, G. H., Frimurer, T. M. and Olsen, O. H. (1998). Electrostatic evaluation of the signature motif (H/V)CX5R(S/T) in protein-tyrosine phosphatases. *Biochemistry* 37, 5383-5393. 10.1021/bi971187i 9548920

[DMM050169C33] Rahdar, M., Inoue, T., Meyer, T., Zhang, J., Vazquez, F. and Devreotes, P. N. (2009). A phosphorylation-dependent intramolecular interaction regulates the membrane association and activity of the tumor suppressor PTEN. *Proc. Natl. Acad. Sci. USA* 106, 480-485. 10.1073/pnas.0811212106 19114656 PMC2626728

[DMM050169C34] Roth, M. G. (2004). Phosphoinositides in constitutive membrane traffic. *Physiol. Rev.* 84, 699-730. 10.1152/physrev.00033.2003 15269334

[DMM050169C35] Sasaki, T., Takasuga, S., Sasaki, J., Kofuji, S., Eguchi, S., Yamazaki, M. and Suzuki, A. (2009). Mammalian phosphoinositide kinases and phosphatases. *Prog. Lipid Res.* 48, 307-343. 10.1016/j.plipres.2009.06.001 19580826

[DMM050169C36] Sasaki, J., Kofuji, S., Itoh, R., Momiyama, T., Takayama, K., Murakami, H., Chida, S., Tsuya, Y., Takasuga, S., Eguchi, S. et al. (2010). The PtdIns(3,4)P2 phosphatase INPP4A is a suppressor of excitotoxic neuronal death. *Nature* 465, 497-501. 10.1038/nature09023 20463662

[DMM050169C37] Shearn, C. T., Walker, J. and Norris, F. A. (2001). Identification of a novel spliceoform of inositol polyphosphate 4-phosphatase type Iα expressed in human platelets: structure of human inositol polyphosphate 4-phosphatase type I gene. *Biochem. Biophys. Res. Commun.* 286, 119-125. 10.1006/bbrc.2001.5331 11485317

[DMM050169C38] Sheffer, R., Bennett-Back, O., Yaacov, B., Edvardson, S., Gomori, M., Werner, M., Fahham, D., Anteby, I., Frumkin, A., Meiner, V. et al. (2015). Hindbrain malformation and myoclonic seizures associated with a deleterious mutation in the INPP4A gene. *Neurogenetics* 16, 23-26. 10.1007/s10048-014-0428-7 25338135

[DMM050169C39] Sun, Y., Ning, X., Fan, J., Hu, J., Jiang, Y., Hu, Z., Paulo, J. A., Liu, J., Qiu, X., Xu, H. et al. (2020). Loss of tumor suppressor inositol polyphosphate 4-phosphatase type B impairs DNA double-strand break repair by destabilization of DNA tethering protein Rad50. *Cell Death Dis.* 11, 292. 10.1038/s41419-020-2491-3 32341333 PMC7184567

[DMM050169C40] Takebayashi, H., Yoshida, S., Sugimori, M., Kosako, H., Kominami, R., Nakafuku, M. and Nabeshima, Y. (2000). Dynamic expression of basic helix-loop-helix Olig family members: implication of Olig2 in neuron and oligodendrocyte differentiation and identification of a new member, Olig3. *Mech. Dev.* 99, 143-148. 10.1016/S0925-4773(00)00466-4 11091082

[DMM050169C41] Thumkeo, D., Shinohara, R., Watanabe, K., Takebayashi, H., Toyoda, Y., Tohyama, K., Ishizaki, T., Furuyashiki, T. and Narumiya, S. (2011). Deficiency of mDia, an actin nucleator, disrupts integrity of neuroepithelium and causes periventricular dysplasia. *PLoS One* 6, e25465. 10.1371/journal.pone.0025465 21980468 PMC3182227

[DMM050169C42] Vanhaesebroeck, B., Guillermet-Guibert, J., Graupera, M. and Bilanges, B. (2010). The emerging mechanisms of isoform-specific PI3K signalling. *Nat. Rev. Mol. Cell Biol.* 11, 329-341. 10.1038/nrm2882 20379207

[DMM050169C43] Volpatti, J. R., Al-Maawali, A., Smith, L., Al-Hashim, A., Brill, J. A. and Dowling, J. J. (2019). The expanding spectrum of neurological disorders of phosphoinositide metabolism. *Dis. Model. Mech.* 12, dmm038174. 10.1242/dmm.038174 31413155 PMC6737944

[DMM050169C44] Waugh, M. G. (2015). PIPs in neurological diseases. *Biochim. Biophys. Acta* 1851, 1066-1082. 10.1016/j.bbalip.2015.02.002 25680866

[DMM050169C46] Wu, T., Yin, F., Guang, S., He, F., Yang, L. and Peng, J. (2020). The Glycosylphosphatidylinositol biosynthesis pathway in human diseases. *Orphanet J. Rare Dis.* 15, 129. 10.1186/s13023-020-01401-z 32466763 PMC7254680

[DMM050169C47] Xu, C. and Zhang, J. (2020). Mammalian alternative translation initiation is mostly nonadaptive. *Mol. Biol. Evol.* 37, 2015-2028. 10.1093/molbev/msaa063 32145028 PMC7828576

[DMM050169C48] Yang, J., Wu, Z., Renier, N., Simon, D. J., Uryu, K., Park, D. S., Greer, P. A., Tournier, C., Davis, R. J. and Tessier-Lavigne, M. (2015). Pathological axonal death through a MAPK cascade that triggers a local energy deficit. *Cell* 160, 161-176. 10.1016/j.cell.2014.11.053 25594179 PMC4306654

[DMM050169C49] Yoshioka, N., Kabata, Y., Kuriyama, M., Bizen, N., Zhou, L., Tran, D. M., Yano, M., Yoshiki, A., Ushiki, T., Sproule, T. J. et al. (2020). Diverse dystonin gene mutations cause distinct patterns of *Dst* isoform deficiency and phenotypic heterogeneity in *Dystonia musculorum* mice. *Dis. Model. Mech.* 13, dmm041608. 10.1242/dmm.041608 32482619 PMC7325434

[DMM050169C50] Yoshioka, N., Kurose, M., Yano, M., Tran, D. M., Okuda, S., Mori-Ochiai, Y., Horie, M., Nagai, T., Nishino, I., Shibata, S. et al. (2022). Isoform-specific mutation in Dystonin-b gene causes late-onset protein aggregate myopathy and cardiomyopathy. *Elife* 11, e78419. 10.7554/eLife.78419 35942699 PMC9365387

[DMM050169C51] Zhang, S.-X., Duan, L.-H., He, S.-J., Zhuang, G.-F. and Yu, X. (2017). Phosphatidylinositol 3,4-bisphosphate regulates neurite initiation and dendrite morphogenesis via actin aggregation. *Cell Res.* 27, 253-273. 10.1038/cr.2017.13 28106075 PMC5339852

[DMM050169C52] Zhou, L., Hossain, M. I., Yamazaki, M., Abe, M., Natsume, R., Konno, K., Kageyama, S., Komatsu, M., Watanabe, M., Sakimura, K. et al. (2018). Deletion of exons encoding carboxypeptidase domain of Nna1 results in Purkinje cell degeneration (*pcd*) phenotype. *J. Neurochem.* 147, 557-572. 10.1111/jnc.14591 30225910

[DMM050169C53] Zhou, L., Konno, K., Yamazaki, M., Abe, M., Natsume, R., Watanabe, M., Takebayashi, H. and Sakimura, K. (2022). Nna1, essential for Purkinje cell survival, is also associated with emotion and memory. *Int. J. Mol. Sci.* 23, 12961. 10.3390/ijms232112961 36361749 PMC9654422

[DMM050169C54] Zolles, G., Klöcker, N., Wenzel, D., Weisser-Thomas, J., Fleischmann, B. K., Roeper, J. and Fakler, B. (2006). Pacemaking by HCN channels requires interaction with phosphoinositides. *Neuron* 52, 1027-1036. 10.1016/j.neuron.2006.12.005 17178405

