# Supplementary material for: Attenuated cerebellar phenotypes in Inpp4a truncation mutants with preserved phosphatase activity
Source: Dis Model Mech. 2023 Jul 28;16(7):dmm050169. doi: 10.1242/dmm.050169 (PMC10399444; doi:10.1242/dmm.050169)
Supplement: Supplementary information [file dmm-16-050169-s1.pdf]

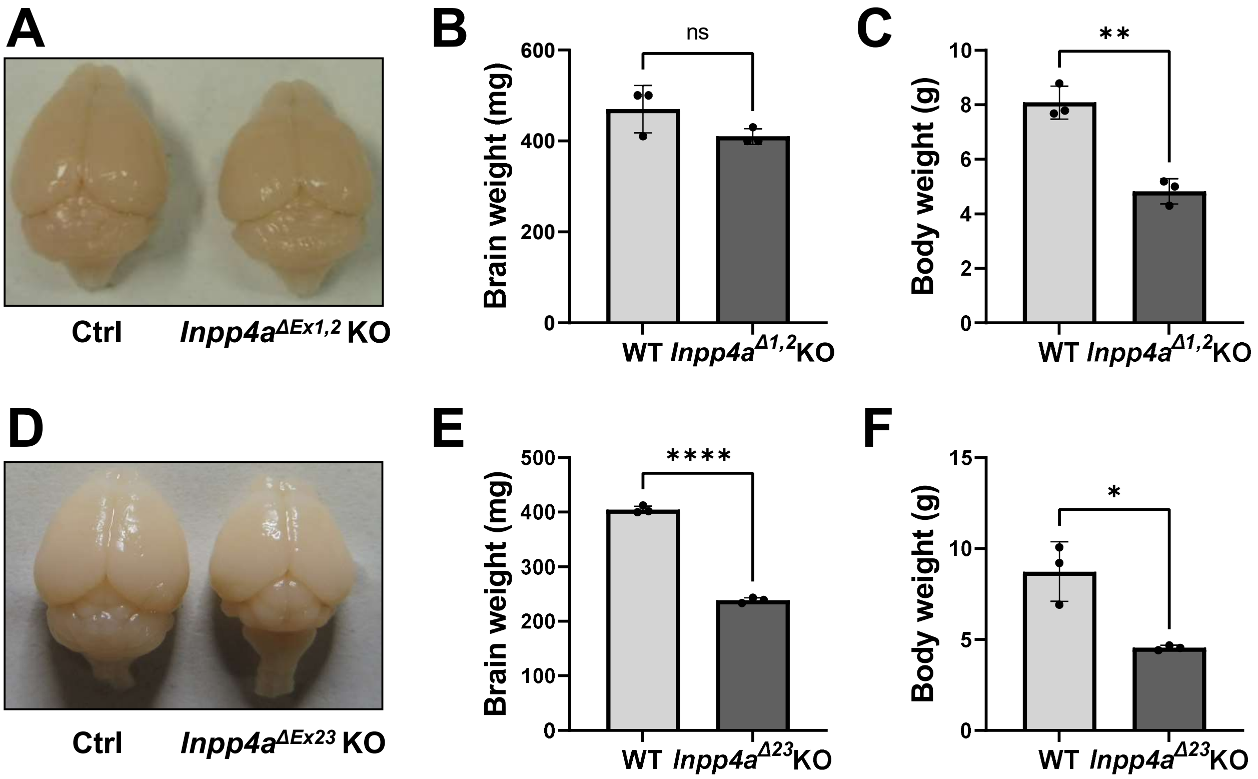

Fig. S1. Apparent cerebellar atrophy in the *Inpp4a*$^{\Delta Ex23}$ KO mice.

(A–C) Comparison between control (Ctrl) and *Inpp4a*$^{\Delta Ex1,2}$ KO brain. Brain picture (A), Brain weight (B), and body weight (C). **(D–F)** Comparison between Ctrl and *Inpp4a*$^{\Delta Ex23}$ KO brain. Brain picture (D), Brain weight (E), and body weight (F). The graphs present all values as means ± SD from 3 mice in each genotype. Statistical significance was set at a value of *$p<0.05$, **$p<0.01$, ****$p<0.0001$ (unpaired two-tailed Student's t-test). “ns” means not significant.

Disease Models & Mechanisms • Supplementary information

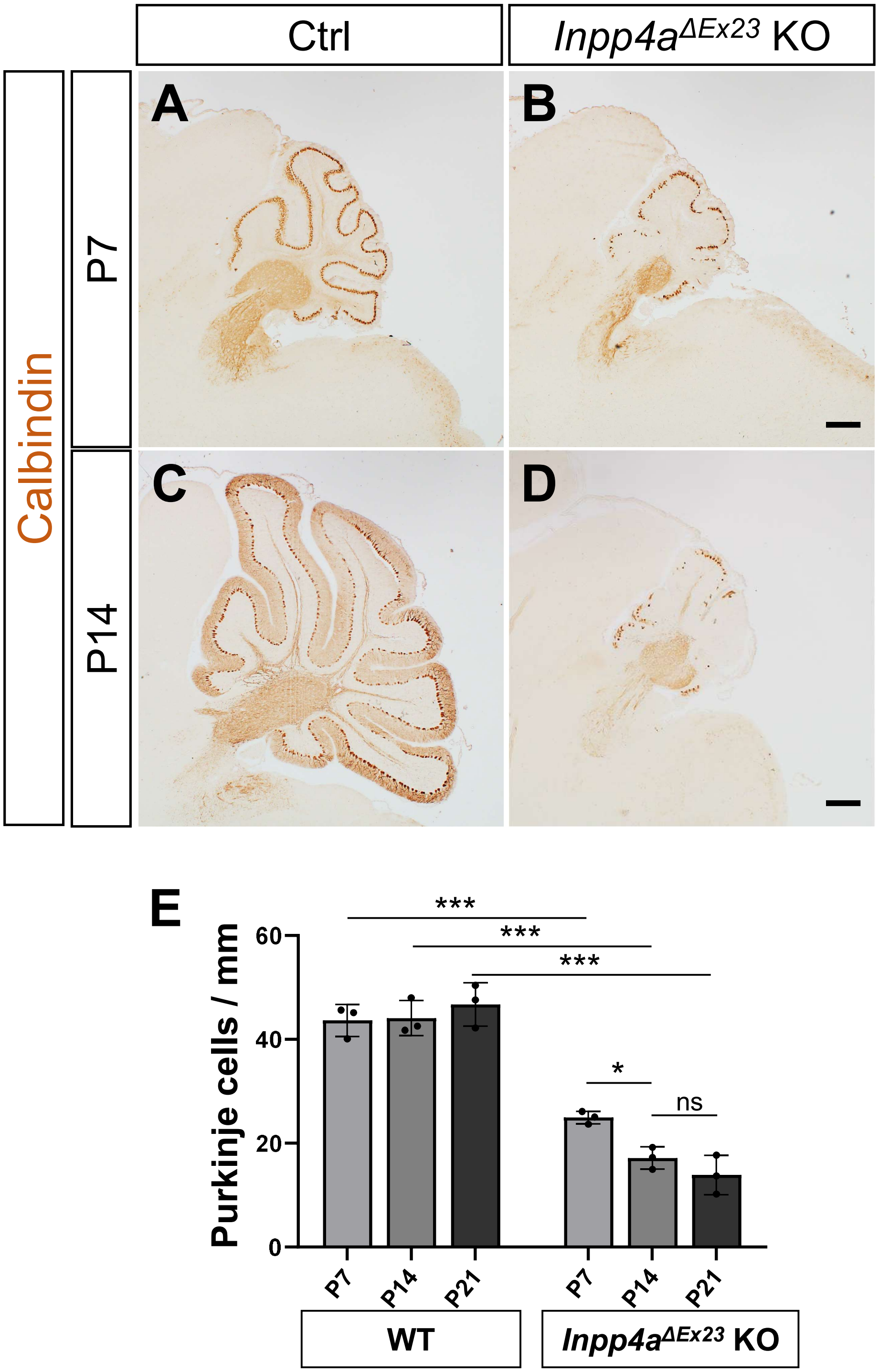

Fig. S2. Postnatal cerebellar degeneration in *Inpp4a*$^{\Delta Ex23}$ KO mice.

(A–D) Calbindin IHC on the parasagittal cerebellum sections of Ctrl and *Inpp4a*$^{\Delta Ex23}$ KO mice at 1 weeks old (A–B) and 2 weeks old (C–D) (n=3 mice, each genotype). **(E)** Quantification of Calbindin-positive cell number (n=3 mice, each genotype). The numbers were normalized by the length of Purkinje cells layer. The graphs present all values as means ± SD from 3 mice in each genotype. Statistical significance was set at a value of *p<0.05, ***p<0.001 (1-way ANOVA). “ns” means not significant. Scale bars: 200 µm.

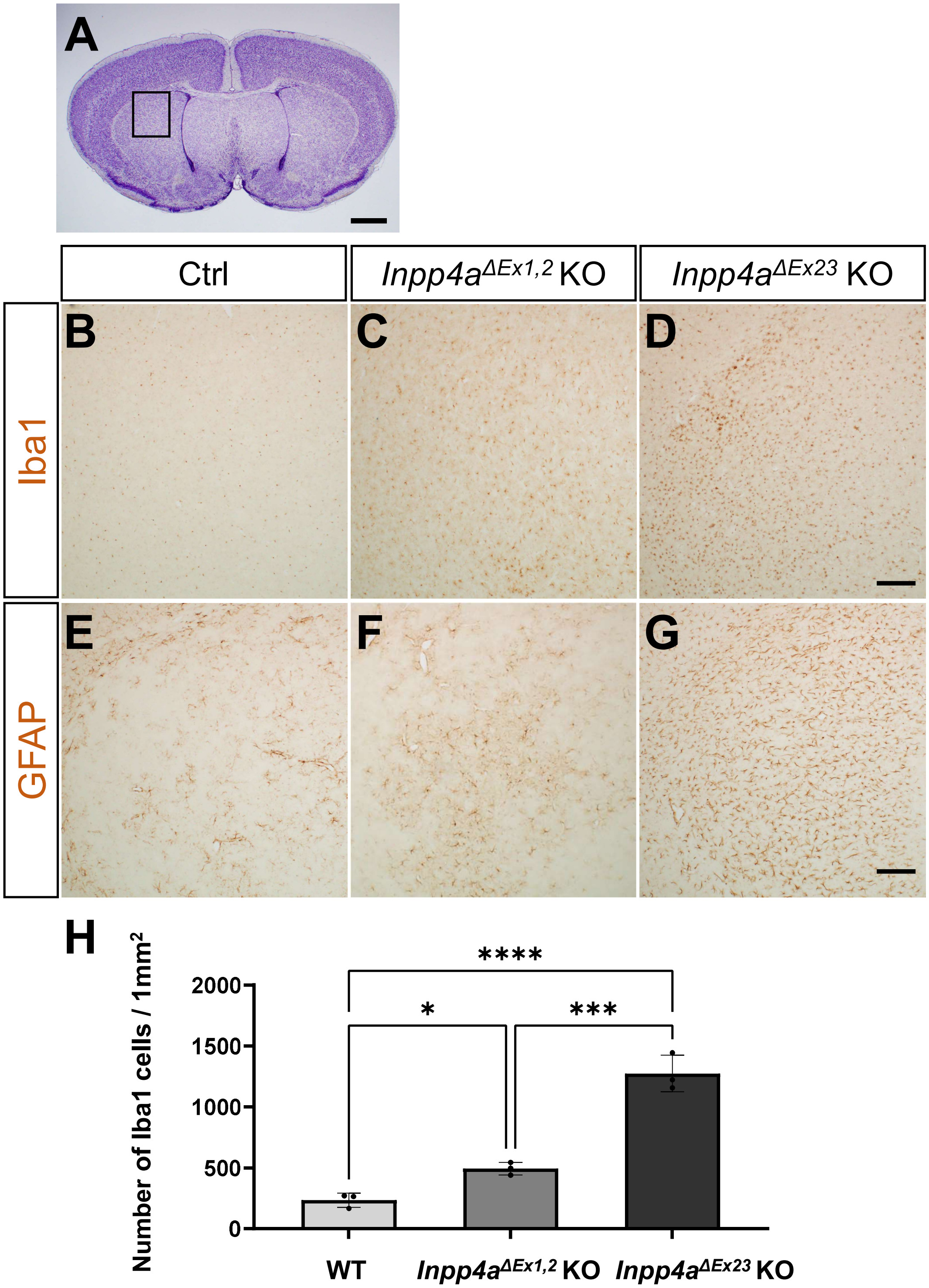

Fig. S3. Striatal phenotypes of *Inpp4a*$^{\Delta Ex1,2}$ KO and *Inpp4a*$^{\Delta Ex23}$ KO mice.

(A) Nissl staining of coronal sections of wild-type brain at 3 weeks old. **(B–D)** Iba1 IHC of control (Ctrl) (B), *Inpp4a*$^{\Delta Ex1,2}$ KO (C), and *Inpp4a*$^{\Delta Ex23}$ KO mice (D) at 3 weeks old (n=3 mice, each genotype). **(E–G)** GFAP IHC of Ctrl (E), *Inpp4a*$^{\Delta Ex1,2}$ KO (F), and *Inpp4a*$^{\Delta Ex23}$ KO mice (G) at 3 weeks old (n=3 mice, each genotype). **(H)** Quantification of Iba1-positive cell number (n=3 mice, each genotype). The graphs present all values as means ± SD from 3 mice in each genotype. Statistical significance was set at a value of *p<0.05, ***p<0.001, ****p<0.0001 (1-way ANOVA). Scale bars: 1 mm (A), 100 μm (B–G).

Disease Models & Mechanisms • Supplementary information

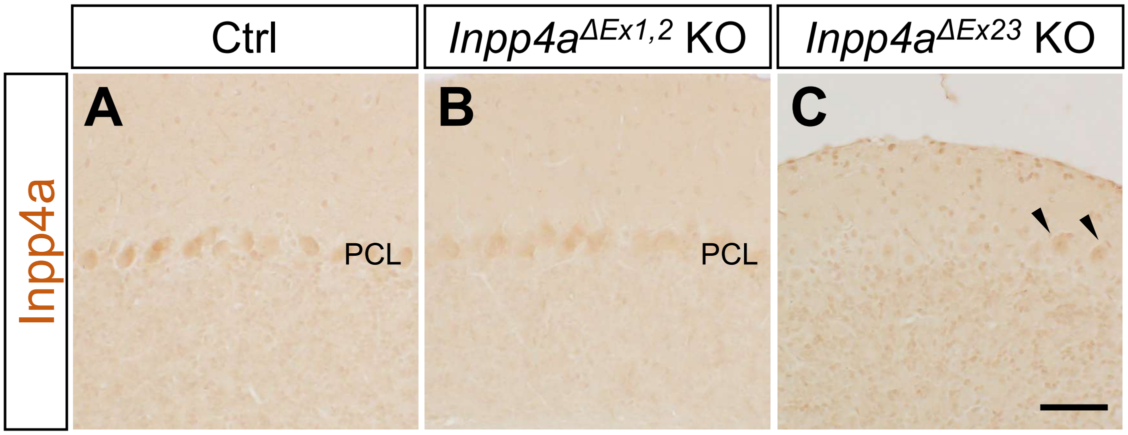

Fig. S4. Inpp4a expression in the Purkinje cells in the *Inpp4a$^{\Delta Ex1,2}$* KO and *Inpp4a$^{\Delta Ex23}$* KO mice.

(A–C) Inpp4a IHC of control (Ctrl) (A), *Inpp4a$^{\Delta Ex1,2}$* KO (B), and *Inpp4a$^{\Delta Ex23}$* KO mice (C) at 3 weeks old (n=3 mice, each genotype). Inpp4a-positive Purkinje cells were observed in the Purkinje cell layers. Arrowheads indicate Inpp4a-positive remaining Purkinje cells in the *Inpp4a$^{\Delta Ex23}$* KO cerebellum. PCL: Purkinje cell layer. Scale bars: 50 μm.

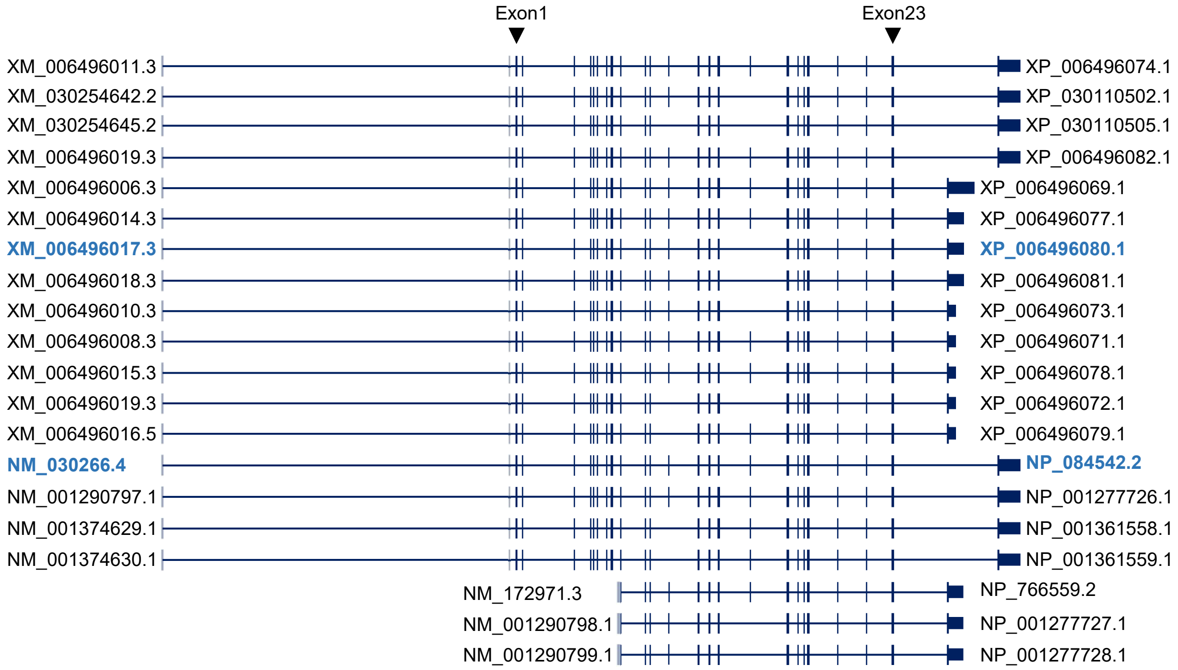

Fig. S5. Diagrammatic view of different types of mouse *Inpp4a* transcripts.

There are several types of mouse *Inpp4a* transcripts deposited in the NCBI database (https://www.ncbi.nlm.nih.gov). NM_030266.4 and NP_084542.2 are the accession numbers for the mRNA and protein isoforms of Inpp4a alpha, respectively. XM_006496017.3 and XP_006496080.1 are the accession numbers for the mRNA and protein isoforms of Inpp4a beta, respectively.

Disease Models & Mechanisms • Supplementary information

Table S1. Human *INPP4A* mutant alleles present in dbSNP database and literatures.

No	dbSNP ID	*INPP4A* exon	Base substitution	Amino acid substitution	Condition(s)	Clinical significance (refenerece)	ClinVar Accession
1	1574887674	Exon 2	NM_001134225.2:c.115C>T	p.Gln39Ter	Intellectual disability	Pathogenic (*Eur J Med Genet* 63, 103846, 2020)	VCV000801338
2	866060075	Exon 2	NM_001134225.2:c.127G>T	p.Glu43Ter			
3	1382098406	Exon 2	NM_001134225.2:c.137del	p.Ile45_Leu46insTer			
4	777176771	Exon 3	NM_001134225.2:c.184C>T	p.Arg62Ter			
5	1574965220	Exon 4	NM_001134225.2:c.352_353del	p.Ser118fs	Microcephaly|visual disturbance|Nystagmus|Pectus excavatum|Hypotonia	Likely pathogenic	VCV000804386
6	1247867306	Exon 4	NM_001134225.2:c.379C>T	p.Gln127Ter			
7	1559033620	Exon 7	NM_001134225.2:c.625C>T	p.Arg209Ter			
8		Exon 7	NM_001134225.2:c.646C > T	p.Arg216Ter	Pontocerebellar hypoplasia | Myoclonic epilepsy| Microcephaly	Pathogenic (*Brain Dev* 45, 300-305, 2023)	
9	1690863098	Exon 7	NM_001134225.2: c.662_663insATTGTGAATA	p.Lys222_Ser223insLeuTer			
10	1422251317	Exon 12	NM_004027.3:c.1168G>A	p.Glu390Ter			
11	1227700629	Exon 14	NM_001134225.2:c.1528C>T	p.Arg510Ter			
12	754812282	Exon 14	NM_001134225.2:c.1565G>A	p.Trp522Ter			
13	1473289811	Exon 15	NM_001134225.2:c.1577G>A	p.Trp526Ter			
14		Exon15	NM_001134225.2: c.1581 del256	p.Glu528Ilefs*22	Hindbrain malformation | Myoclonic seizures	Pathogenic (*Neurogenetics* 16, 23–26, 2015)	
15	774856300	Exon 15	NM_001134225.2:c.1624A>T	p.Lys542Ter			
16	753017989	Exon 15	NM_001134225.2:c.1803C>A	p.Cys601Ter			
17	1413047925	Exon 17	NM_001134225.2:c.2001C>G	p.Tyr667Ter			
18	1696311463	Exon 19	NM_001134225.2:c.2227C>T	p.Gln743Ter			
19	867332311	Exon 22	NM_001134225.2:c.2619G>A	p.Trp873Ter			
20	1198219052	Exon 24	NM_001566.2:c.2740C>T	p.Arg914Ter			
21		Exon 24	NM_001566.2:c.2744del	p.Asp915Alafs*2	Recessive cognitive disorders	Pathogenic (*Nature* 478, 57–63, 2011)	
22	1370549873	Exon 25	NM_001134225.2:c.2797C>T	p.Arg933Ter			
23	2106568683	Exon 25	NM_001134225.2:c.2803G>T	p.Glu935Ter			
24		Exon25	NM_001134224.2:c.2840del	p.Gly947Glufs*12	Microcephaly|Development delay| Myoclonic Seizures | Pontocerebellar hypoplasia	Pathogenic (*Neurogenetics* 24, 79–93, 2023)	
25	2106568821	Exon 25	NM_001134225.2:c.2854C>T	p.Gln952Ter			

The exon 1 is first encoding exon, which contains the ATG encoding the first methionine.

Disease Models & Mechanisms • Supplementary information

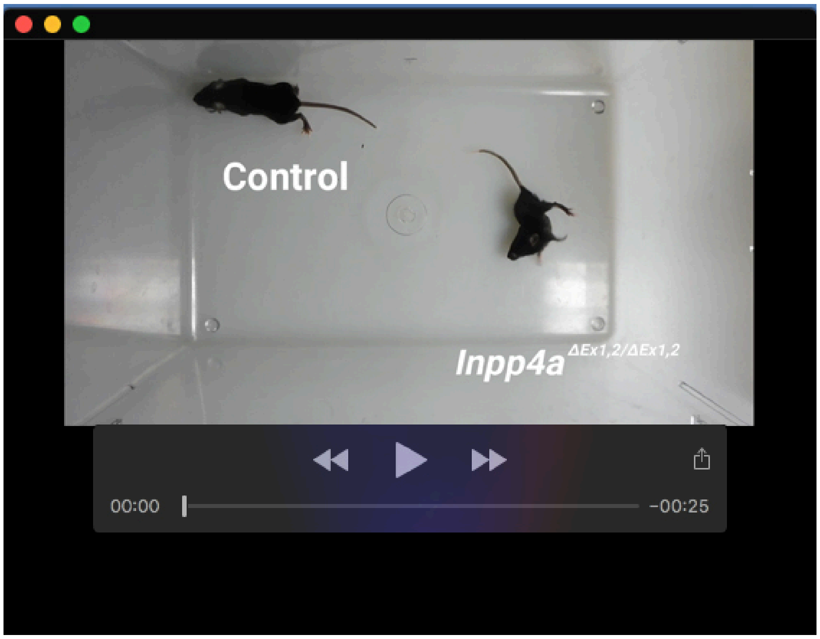

Movie 1. Movement disorder of *Inpp4a*$^{\Delta Ex1,2}$ KO mouse at 3 weeks old.

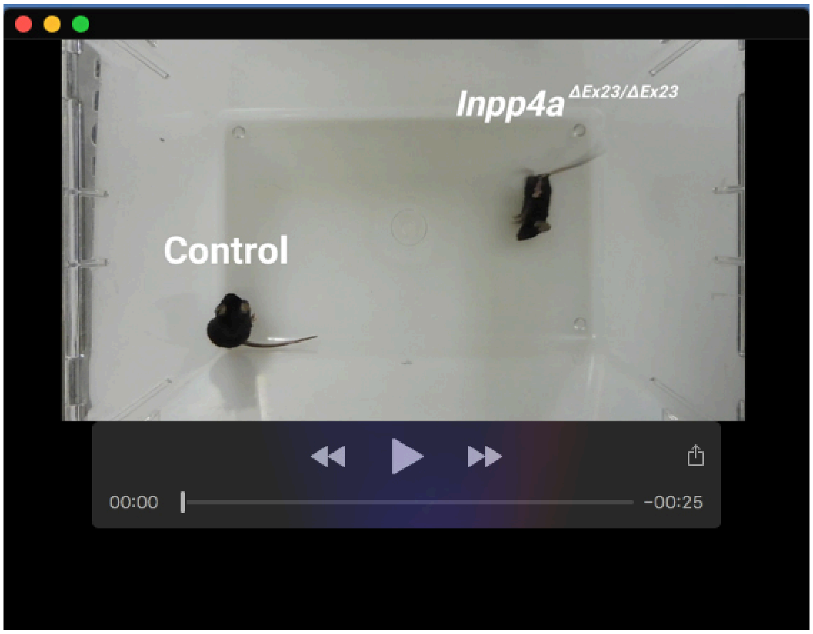

Movie 2. Movement disorder of *Inpp4a*$^{\Delta Ex23}$ KO mouse at 3 weeks old.

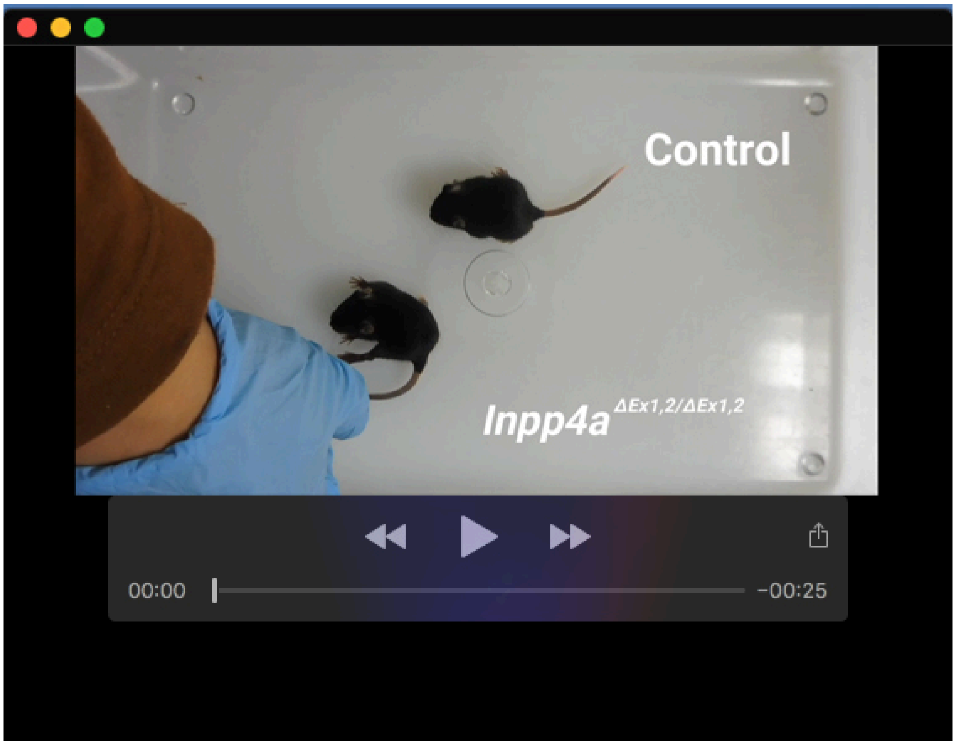

Movie 3. Pain-induced epilepsy in the *Inpp4a*$^{\Delta Ex1,2}$ KO mouse at 3 weeks old.

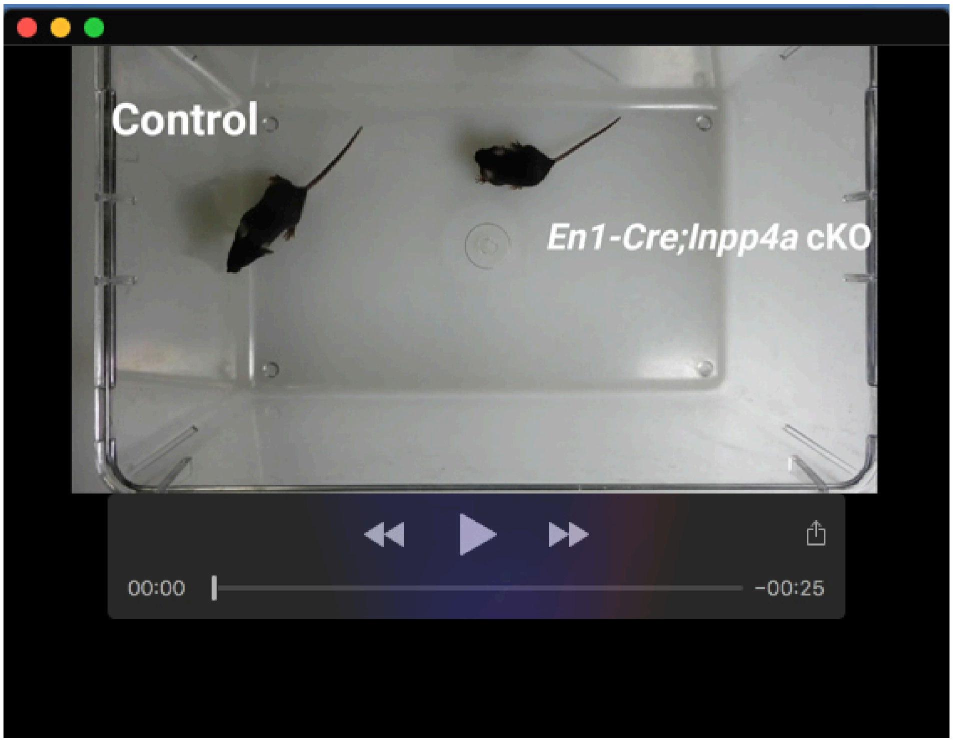

Movie 4. Movement disorder of *En1-Cre;Inpp4a* cKO mouse at 3 weeks old.

Disease Models & Mechanisms • Supplementary information
